# Combination of Ru(ii) complexes and light: new frontiers in cancer therapy

**DOI:** 10.1039/c4sc03759f

**Published:** 2015-01-13

**Authors:** Cristina Mari, Vanessa Pierroz, Stefano Ferrari, Gilles Gasser

**Affiliations:** a Department of Chemistry , University of Zurich , Winterthurerstrasse 190 , CH-8057 Zurich , Switzerland . Email: gilles.gasser@chem.uzh.ch ; http://www.gassergroup.com ; Fax: +41 44 635 6803 ; Tel: +41 44 635 4630; b Institute of Molecular Cancer Research , University of Zurich , Winterthurerstrasse 190 , CH-8057 Zurich , Switzerland

## Abstract

The synergistic action of light, oxygen and a photosensitizer (PS) has found applications for decades in medicine under the name of photodynamic therapy (PDT) for the treatment of skin diseases and, more recently, for the treatment of cancer. However, of the thirteen PSs currently approved for the treatment of cancer over more than 10 countries, only two contain a metal ion. This fact is rather surprising considering that nowadays around 50% of conventional chemotherapies involve the use of cisplatin and other platinum-containing drugs. In this perspective article, we review the opportunities brought by the use of Ru(ii) complexes as PSs in PDT. In addition, we also present the recent achievements in the application of Ru(ii) complexes in photoactivated chemotherapy (PACT). In this strategy, the presence of oxygen is not required to achieve cell toxicity. This is of significance since tumors are generally hypoxic. Importantly, this perspective article focuses particularly on the Ru(ii) complexes for which an *in vitro* biological evaluation has been performed and the mechanism of action (partially) unveiled.

## Abstract 2

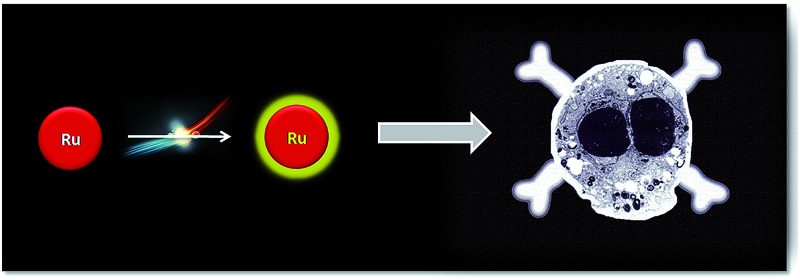
In this perspective article, we present the recent achievements in the application of ruthenium complexes as photosensitizers and as photoactivatable prodrugs.

## Introduction

**Fig. 1:**
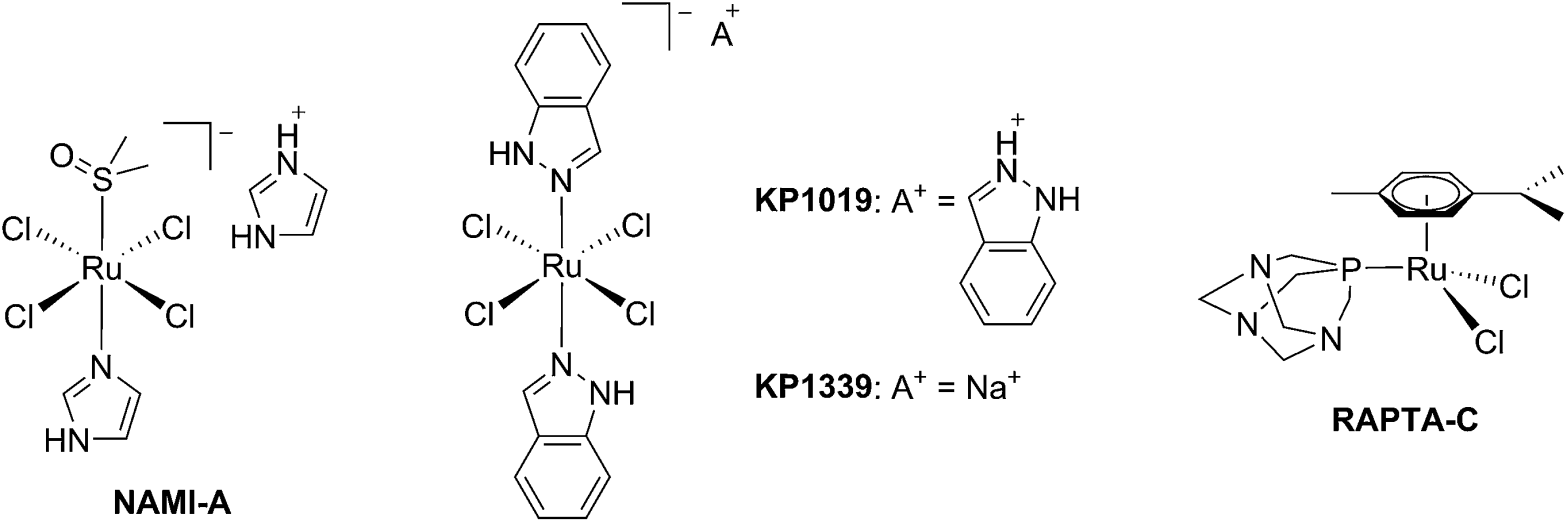
Structures of NAMI-A, KP1339, KP1019 and RAPTA-C.

The biological activity of ruthenium (Ru) compounds has been known for decades.^1–3^ Two Ru complexes are currently in phase II clinical trials (NAMI-A and KP1339) as anticancer drug candidates and a third one, RAPTA-C, is progressing towards clinical trials (see Fig. 1 for the structures of these compounds).^4–10^ The increasing interest in the biological behavior of Ru compounds is due to their appealing physico-chemical properties. Among others, such complexes can have different geometries (*e.g.* tetrahedral or octahedral) allowing for the design of compounds with a specific cellular target (*e.g.* proteins). Hence, the rigid and well-defined spatial arrangement of a series of Ru complexes has enabled the preparation of highly potent and selective enzyme inhibitors. The group of Meggers has notably demonstrated such a concept with kinase inhibitors.^2,11,12^ Other attractive features of Ru complexes include their generally lower systemic toxicity compared to platinum complexes and their higher cellular uptake, thanks to the specific transport of ruthenium inside cells by transferrin.^13^ Of utmost importance, ruthenium complexes can easily be obtained in two oxidation states (ii and iii) and are prone to ligand exchange. Such properties have been found to play a pivotal role in the mode of action of both NAMI-A and KP1339.^14^ Ru(iii) complexes are thus prodrugs – meaning that the compound which is administered to the patient is not the active species. Ru(iii) complexes are reduced into a more active Ru(ii) form when localized in an hypoxic environment, which is a property characteristic of tumors.^6^ This phenomenon is normally referred to as “activation by reduction” and was also exploited for the *in situ* activation of Pt-based anticancer drug candidates, like satraplatin.^15^

Nowadays, the use of the “prodrug approach” is very appealing to reduce the systemic toxicity of a drug candidate.^16^ In order to activate the prodrugs, two different kinds of stimuli can be employed, namely an internal stimulus (reducing cellular conditions, hypoxia, enzymatic reactions, *etc.*) or an external stimulus (magnetic field, temperature, light, *etc.*). The first approach, however, presents a significant disadvantage, in that it completely relies on intracellular parameters. In other words, once the prodrug is injected into the patient, physicians have no more control over the fate of the compound. On the contrary, this is exactly the kind of control that can be achieved using an external stimulus. The latter indeed provides complete *spatial and temporal control* over the generation of the toxic molecule. As of today, the most commonly applied technique to induce the formation of active species is *via* light irradiation.^15,17,18^

The light-mediated activation of prodrugs in the field of anticancer research can be generally divided into two categories: photodynamic therapy (PDT) and photoactivated chemotherapy (PACT). PDT relies mainly on the generation of the toxic reactive oxygen species (ROS) singlet oxygen (^1^O_2_). On the other hand, PACT exploits different mechanisms to induce cell death such as ligand ejection, DNA crosslinking and caging approaches. In this perspective article, we intend to give an overview of recent progress in the application of ruthenium complexes in both PDT and PACT, focusing particularly on those compounds for which an *in vitro* evaluation of the biological activity has been performed and the mechanism of action (partially) unveiled. Notably, these topics have been partially reviewed in the past but an article covering all subjects is, to the best of our knowledge, currently missing.^17–26^

## Ruthenium complexes as photosensitizers in PDT

**Fig. 2:**
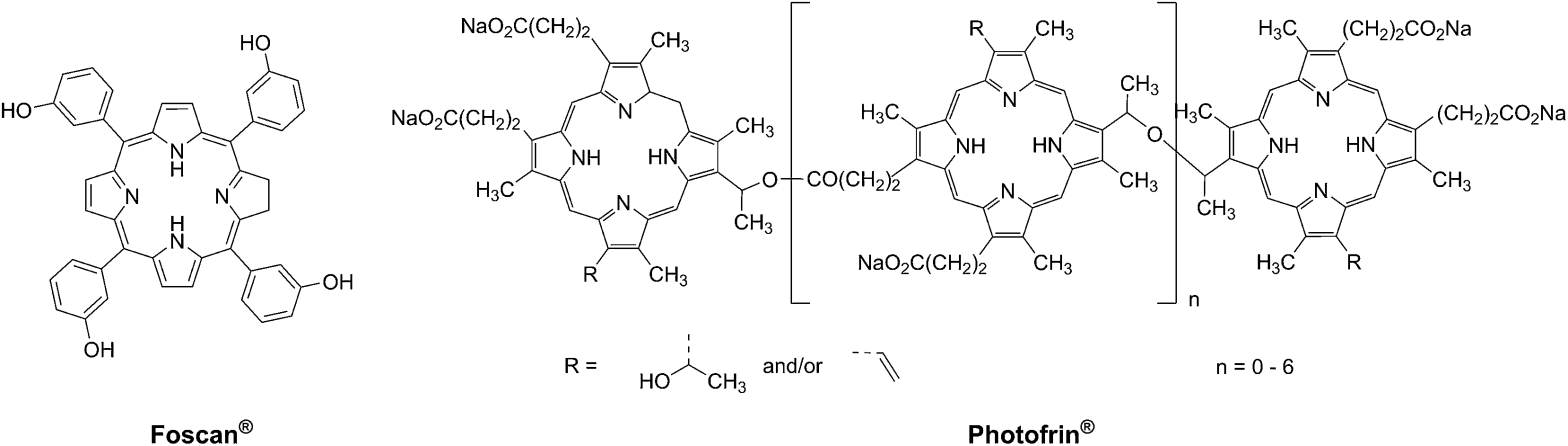
Structures of porphyrin-based approved PDT agents.

Photodynamic therapy is an approved medical technique, which is applied in dermatology for the treatment of several diseases such as acne or psoriasis and in ophthalmology for age-related macular degeneration. Since relatively recently, this technique has been used for the treatment of some types of cancer. For example, Photofrin® (Fig. 2), the only FDA-approved PDT drug, is employed to treat esophageal and non-small cell lung cancers. In the UK, on the other hand, there are several photoactive agents which are clinically approved (*i.e.* Foscan®, Fig. 2) to treat a wide range of cancer types, from skin to internal organs.^27,28^

**Fig. 3:**
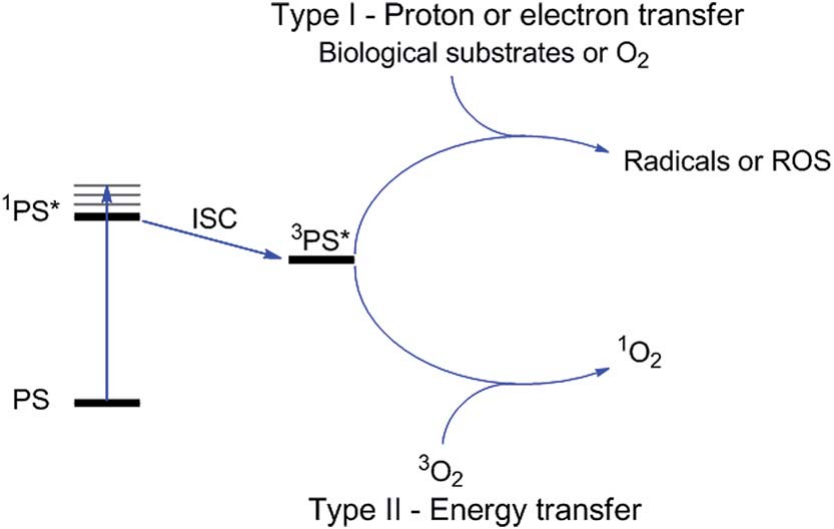
Mechanisms of action of PDT.

More specifically, PDT relies on the synergistic activity of an ideally non-toxic molecule called a photosensitizer (PS), light and molecular oxygen. The PS is administrated to the patient either locally or systemically. Upon light irradiation at a wavelength in its range of absorption, the PS is able to reach its singlet excited state ^1^PS* (Fig. 3). Very importantly, the PS must then undergo an intersystem crossing (ISC) so that the excited state has a triplet character (^3^PS*). At this point, PDT relies on two different mechanisms called Type I and Type II. A Type I reaction consists of an electron or proton transfer from the triplet excited state of the PS to the surrounding biological substrates (or the other way around). This leads to the formation of radicals that can further interact with molecular oxygen to form ROS such as superoxide, hydroxyl radicals or peroxides. At the same time, an energy transfer from the triplet excited state of the PS to molecular oxygen in its ground triplet state (^3^O_2_) can occur (a Type II reaction). In this case, singlet oxygen (^1^O_2_) is generated. ^1^O_2_ is a very reactive form of oxygen with an estimated half life of 40 ns in a biological environment.^27^ Consequently, it will rapidly react just with the surrounding biomolecules, generating topical cellular damage that can ultimately lead to cell death. PSs which are nowadays applied in clinics mainly rely on the Type II mechanism of action.^29^

PDT is a very appealing medical technique due to its intrinsic selectivity. The toxic species are generated just at the site of light irradiation, with complete spatial and temporal control. Furthermore, due to the very fast reactivity of ^1^O_2_, damage is limited to the irradiated areas. The outcomes of PDT treatment depend on the performance of the PS but also on other very important factors (*e.g.* the light component, the *in vivo* dosimetry or the oxygen tension). To be clinically applicable, a PS should, among other requirements, (i) localize mainly (ideally only) in cancer cells; (ii) should be non-toxic in the absence of light, while displaying strong phototoxicity. This behavior is normally described by the so-called phototoxic index (PI), defined for a compound as the ratio of its IC_50_ in the dark to its IC_50_ upon light irradiation. Finally, the PS (iii) should be excited in the red or near-IR region of the spectrum (>600 nm). This last requirement is very important to avoid cytotoxicity deriving from high energy light irradiation. In addition, the use of long wavelength light allows for a deeper penetration through the human tissues.^21,30^

The great majority of PSs that are currently applied in clinics are based on a cyclic tetrapyrrolic scaffold. The photophysical and biological characteristics of porphyrins, phthalocyanines and chlorins match the requirements for a PDT agent relatively well. On the other hand, their performances are also limited by important side-effects. As an example, treatment with Photofrin® results in light sensitivity for several weeks due to slow clearance of the drug from the body.^31^ As a consequence, an important effort has been undertaken to improve the performances of the current PSs following two approaches: the modification of a conventional porphyrin-based PS or the optimization of entirely new systems that can outperform porphyrins in their PDT activity. In this specific section, we present a description of the influence of the insertion of ruthenium fragments into porphyrin-based PSs, focusing our attention on the works that report on the biological behavior of these new systems. Furthermore, we present the recent achievements in the use of ruthenium polypyridyl complexes as novel PSs in the innovative attempt to move away from the traditional porphyrin-as-PS paradigm.

### Ruthenium-containing porphyrin PSs

**Fig. 4:**
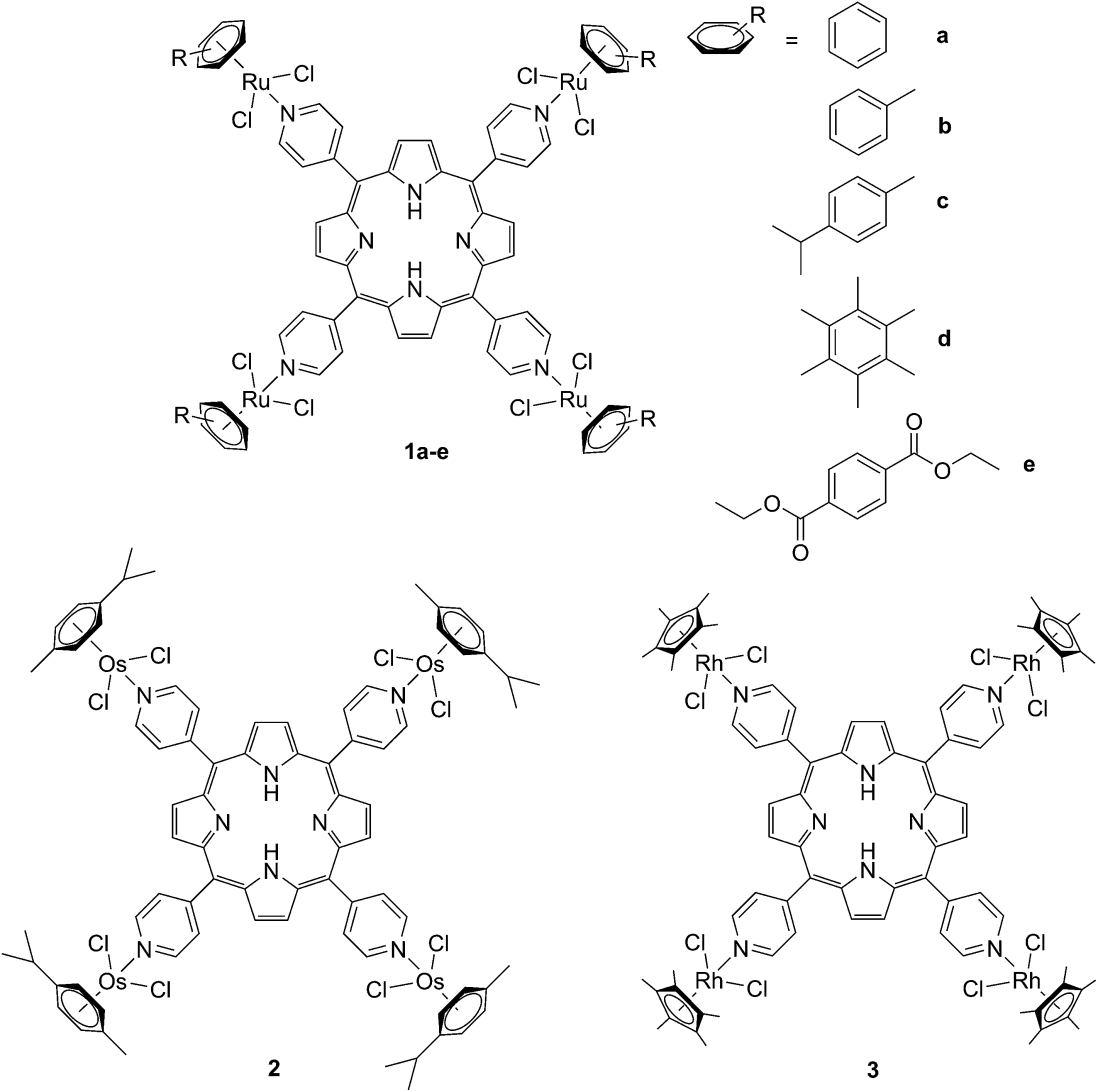
Structures of Ru–porphyrin conjugates (top, **1a–e**), and Os and Rh analogs (bottom, **2** and **3**).^34^

**Fig. 5:**
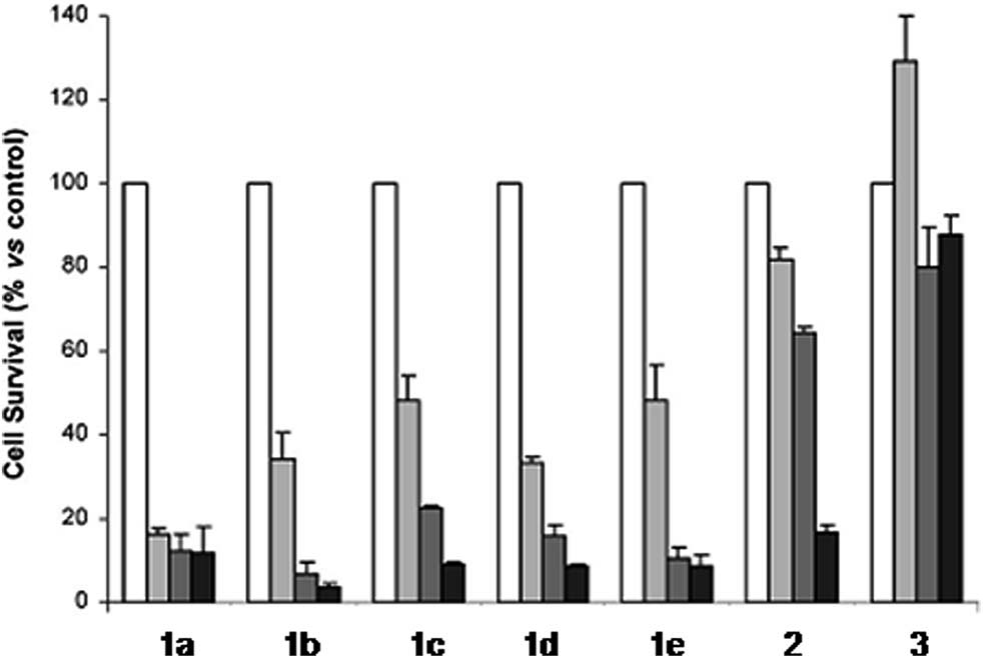
Phototoxicity evaluation of compounds **1a–e**, **2** and **3** on Me300 melanoma cells. Cells were incubated with 10 μM of the compounds, incubated for 24 h, then irradiated at 652 nm with 0 J cm^–2^ (white bar), 5 J cm^–2^ (light grey bar), 15 J cm^–2^ (dark grey bar) or 30 J cm^–2^ (black bar) light doses. Adapted with permission from ref. 34. Copyright 2008 American Chemical Society.

The derivatization of the porphyrin core with metal complexes is an appealing opportunity to improve the activity of a PS. This functionalization was exploited for the first time fifteen years ago by Brunner and coworkers.^32,33^ They synthesized hematoporphyrin–platinum conjugates to combine the strong anticancer activity of platinum-based drugs with the phototoxic effect of porphyrins. The metal derivatization of a porphyrin core can enhance the intrinsic properties of a PS by modifying its physico-chemical characteristics. For example, the metal fragment can change the lipophilicity of the PS, increase its water solubility or improve its cellular uptake. As mentioned above, ruthenium complexes display very promising biological behavior. Consequently, several research groups have recently evaluated the possibility of introducing Ru(ii) moieties on the periphery of porphyrins. For instance, Therrien *et al.* synthesized a wide range of Ru-modified porphyrin systems and studied their biological performances.^34^ More specifically, they appended a number of Ru-arene fragments to the *meso*-4′-tetrapyridylporphyrin scaffold to evaluate the influence of the different aromatic moieties (**1a–e**, Fig. 4, top). All the compounds were found to induce 60–80% mortality in human Me300 melanoma cells at a 10 μM concentration, using light at 652 nm with a dose of 5 J cm^–2^. The photoactivity of the metal-functionalized systems was found to be independent of the nature of the arene. This flexibility can give access to the use of arenes which are derivatized with targeting agents or chemotherapeutic compounds. Fig. 5, which shows the phototoxicity evaluation of the compounds synthesized by Therrien *et al.*, demonstrates that the improved behavior of their systems required the presence of the Ru fragment, since the Rh analog **3** was not internalized by cells and was therefore not toxic. In addition, the Os derivative **2** exerted just a weak phototoxic effect (see Fig. 4 for the structures of the latter compounds).

**Fig. 6:**
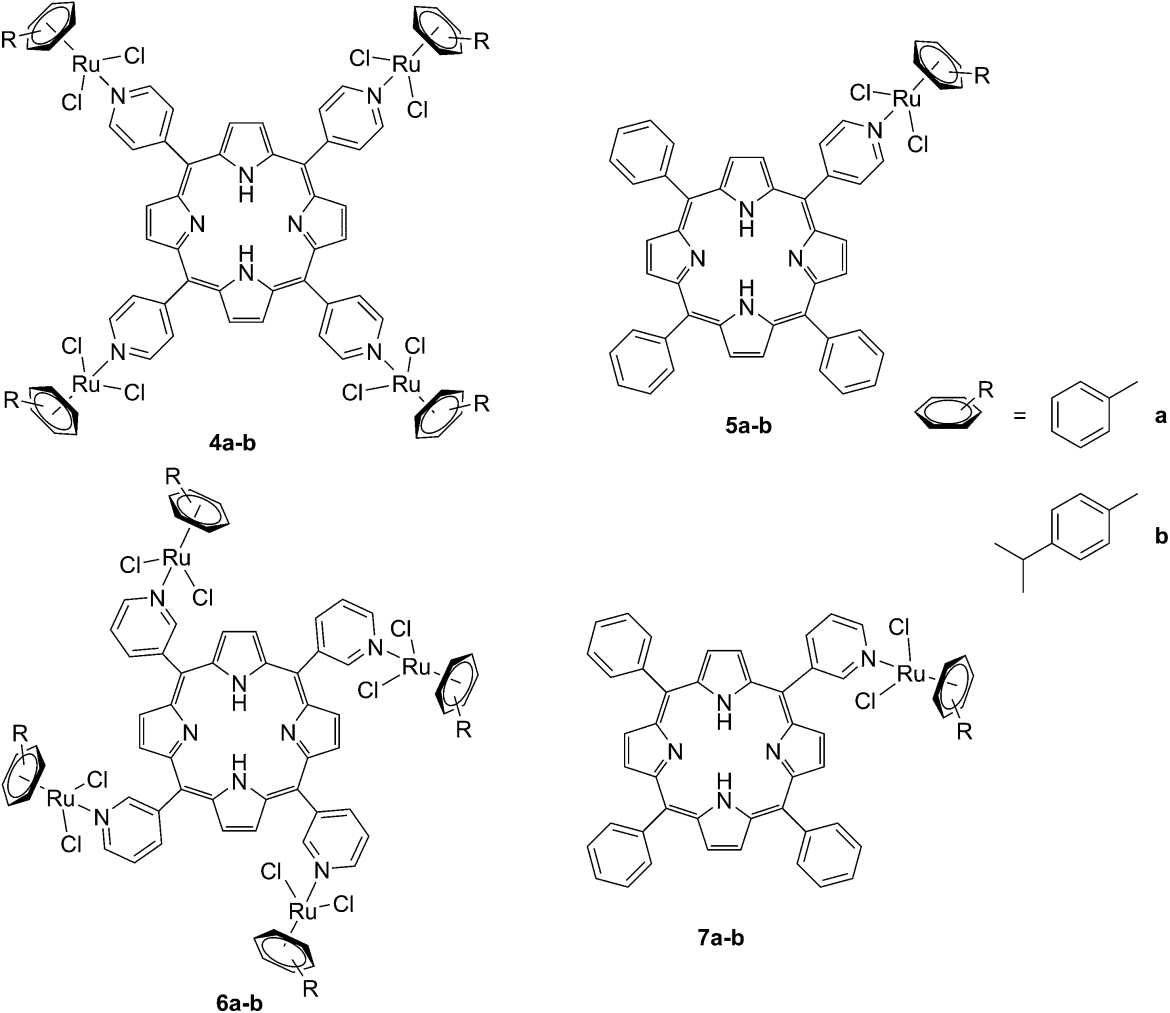
Structures of the Ru–porphyrin conjugates evaluated in the SAR study by Schmitt *et al*.^35^

The same authors also studied the influence of tetra- *vs.* mono-metallic derivatization (**4a-b/6a–b** *vs.* **5a–b/7a–b**, Fig. 6), as well as the nature of the pyridylporphyrin isomers, by comparing 4′-pyridylporphyrin or 3′-pyridylporphyrin derivatives (**4a–b/5a–b** *vs.* **6a–b/7a–b**, Fig. 6).^35^ Several conclusions could be drawn from this small structure–activity relationship (SAR) study. For example, the type of pyridylporphyrin isomer was shown to play a major role in the observed activity, since the 3′-pyridyl substituted compounds showed a greater phototoxic effect than the 4′-pyridyl analogs. On the other hand, the number of Ru atoms or the arene derivatization seemed to have less influence on the biological activity.

**Fig. 7:**
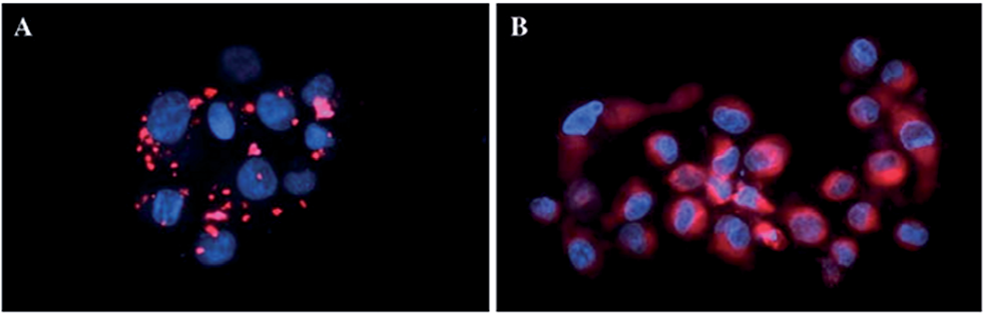
Fluorescence microscopy images of human Me300 melanoma cells incubated for 24 h with 5 μM of **4a** (A) and **6a** (B), displaying red luminescence. The blue luminescence in the nuclei derives from DAPI co-staining. With kind permission from Springer Science and Business Media.^35^

In more detail, upon the 652 nm light irradiation of human Me300 melanoma cells, a LD_50_ of 5 μM was reached with a light dose of 0.5 J cm^–2^ for compounds **6a** and **6b** and with a light dose of 2.5 J cm^–2^ for **7a** and **7b**. For the 4-pyridyl derivatives, 5 or even 10 J cm^–2^ were necessary to achieve the same potency. This difference in biological activity was explained by luminescence microscopy studies, where **4a** (more hydrophobic) was shown to form aggregates inside the cytoplasm (Fig. 7A), although the authors did not discuss further about accumulation in a specific organelle. This aggregation could lead to a quenching of the ROS production. On the contrary, compound **6a** was shown to be evenly distributed in the cytoplasm, where it could exert its phototoxic activity (Fig. 7B).

The two best compounds in this study, namely [Ru(η^6^-*p*-^i^PrC_6_H_4_Me)(PMP)Cl_2_] (PMP = 5-(3-pyridyl)-10,15,20-triphenylporphyrin) and [Ru_4_(η^6^-*p*-^i^PrC_6_H_4_Me)_4_(PTP)Cl_8_] (PTP = 5,10,15,20-tetra(3-pyridyl)porphyrin) (**6b** and **7b**) were evaluated *in vivo* on nude mice xenografted with human head and neck carcinoma KB cells.^36^ Since PDT is a synergistic cooperation of different components (PS, light and O_2_), the evaluation of its *in vivo* efficacy depends on the combination of a complex system of parameters, which reciprocally affect each other. As a consequence, the authors determined that crucial factors to be optimized during *in vivo* studies were not just the concentration of the drug, but also the interval between PS administration and light treatment (the drug-light interval, DLI), light fluence and the fluence rate.^36^ They therefore adopted a statistical approach to find the combination of parameters that would yield the best therapeutic outcomes, thereby reducing as much as possible the number of required experiments. The study showed that, if PS concentration and light fluence were not crucial parameters, a long DLI and the use of the tetranuclear species led to statistically significant tumor growth stabilization up to at least 30 days.

**Fig. 8:**
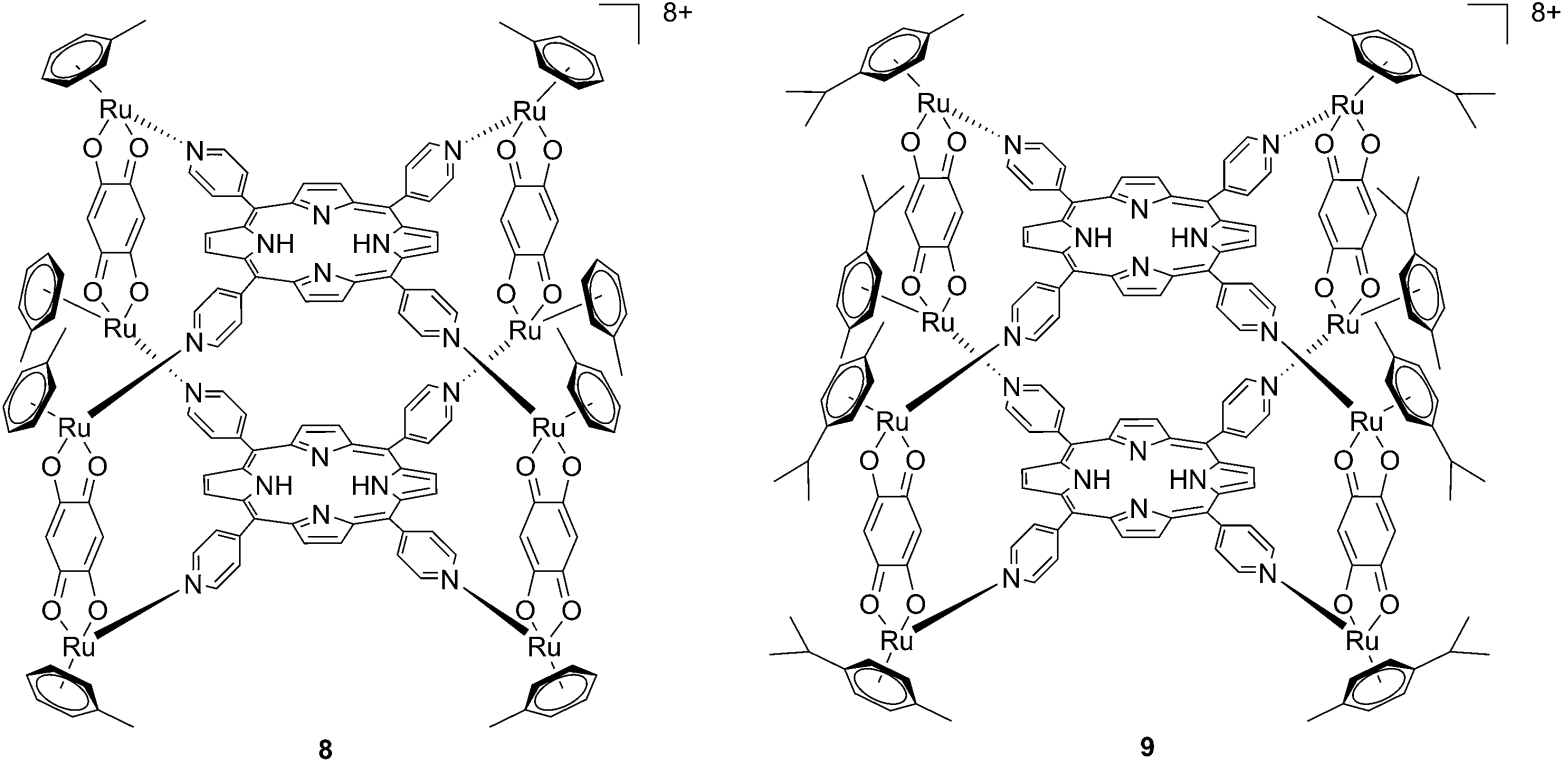
Polynuclear metalla-cubes **8** and **9** synthesized by Therrien to increase phototoxicity.^37^

Since the study on these systems highlighted that the number of ruthenium modifications is correlated with an increase in phototoxicity, the authors synthesized two cationic octanuclear metalla-cubes **8** and **9** (Fig. 8). These compounds, thanks to their higher ruthenium content, showed better activities when compared to their tetranuclear analogs.^37^ An LD_50_ of 1 μM was reached upon irradiation with 652 nm light and a 2–7 J cm^–2^ light dose for both compounds, whereas for the tetranuclear analogs, a light dose of 5–10 J cm^–2^ at the same wavelength resulted in a LD_50_ of 5 μM.

**Fig. 9:**
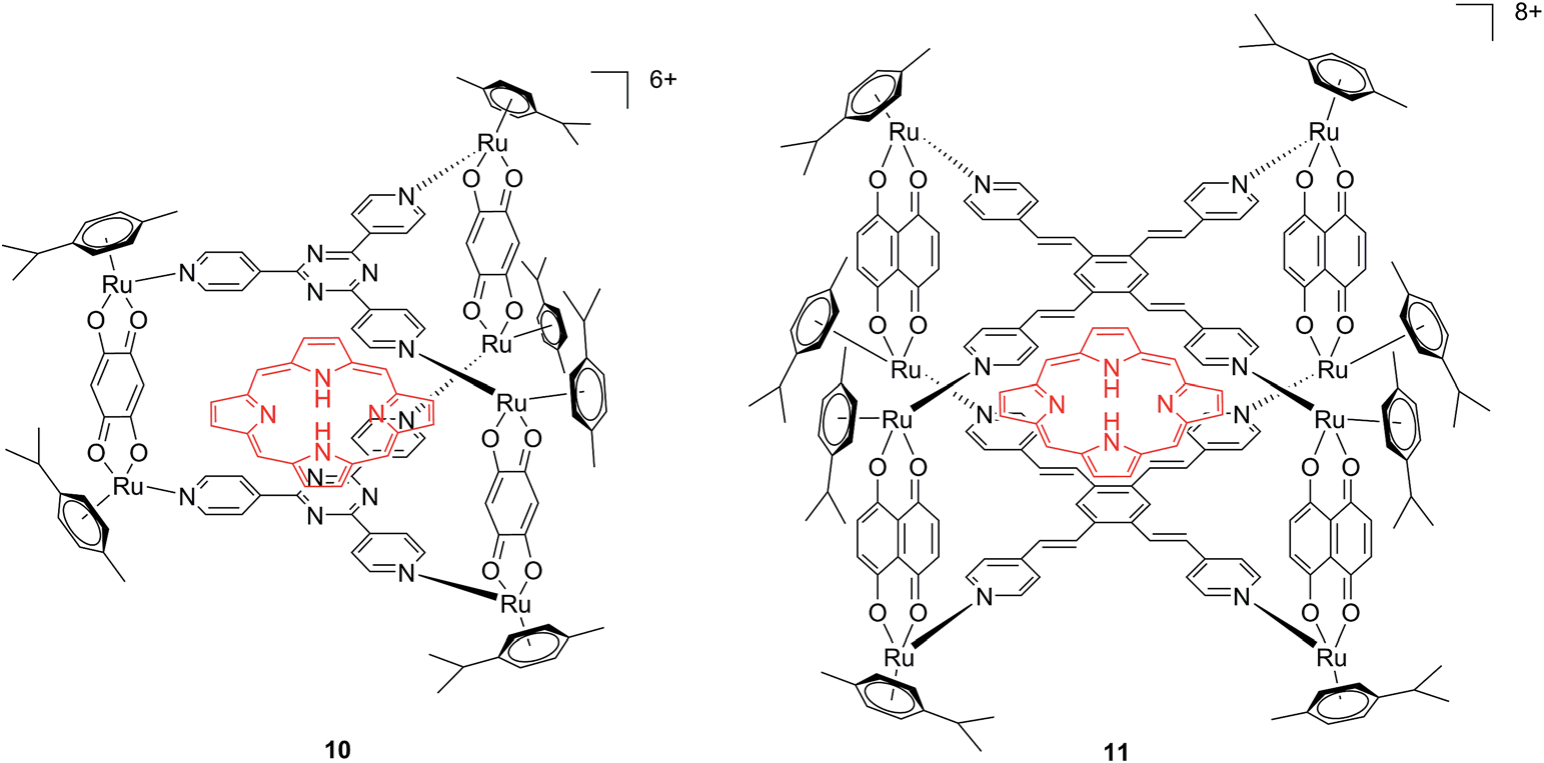
Ruthenium cages **10** and **11** applied as carriers of a porphyrin PS inside cancer cells.^38^

**Fig. 10:**
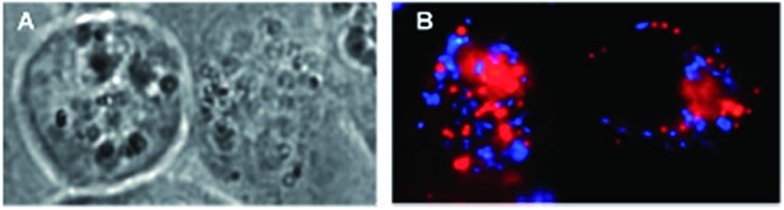
Fluorescence microscopy of HeLa cells incubated with **11** (2 μM, 2 h): (A) white light and (B) fluorescence. Reprinted with permission from ref. 38. Copyright 2012 American Chemical Society.

Another interesting approach used by this group for the combination of Ru complexes and PDT is the application of Ru-cages as carriers for porphyrin photosensitizers inside cancer cells. The authors developed the two cages presented in Fig. 9, namely hexa- (**10**) and octanuclear (**11**), which were characterized by different mechanisms of release.^38^ In the case of **10**, the cage must be disrupted to allow the release of the PS, whereas for **11**, the PS can diffuse through the sides of the cage. As a consequence of this difference, **11** was found to be 10 times more photoactive than **10**. The authors obtained phototoxicity in the submicromolar range and a PI of about 20 for **11** on cervical cancer HeLa cells, upon irradiation at 455 nm with an impressively weak light dose (0.2 J cm^–2^). This result demonstrated the release of the porphyrin after cellular internalization, as was also shown by luminescence microscopy (Fig. 10). In these pictures, it is possible to notice the red luminescence from the free PS and the blue emission originating from the empty cage. This also indicates that the two systems are localizing in different cellular compartments after release. Furthermore, the internalization of the porphyrin in both cages resulted in a hypochromic effect on the porphyrin. This means that when the PS is trapped, its emission is dramatically reduced and consequently also the phototoxic effect. This phenomenon leads to a safe delivering agent that does not display undesired phototoxicity outside of cells.

**Fig. 11:**
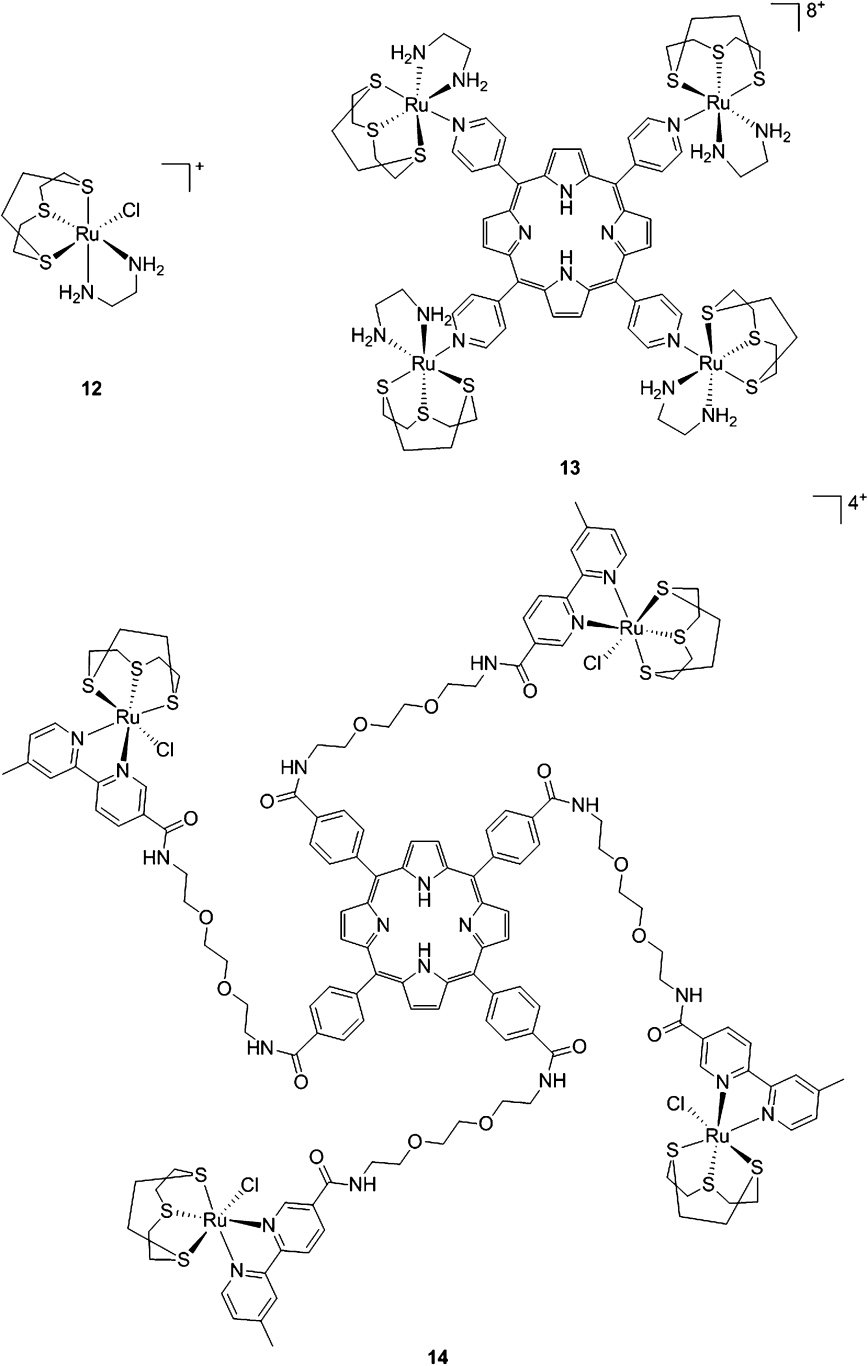
Structures of Ru([9]aneS_3_)(en)Cl]^+^ (top, left) and of the ruthenium-derivatized porphyrin systems **13** and **14**.^40^

With the same idea in mind, namely to obtain a synergistic biological effect owing to the conjugation of porphyrin and ruthenium fragments, Alessio and coworkers synthesized a library of compounds where *meso*-tetraphenylporphyrin or *meso*-4′-tetrapyridylporphyrin cores were modified on their peripheries with Ru complexes.^39^ The authors then selected five cationic species for biological evaluation.^40^ The most active compounds **13** and **14** (Fig. 11) contain four ruthenium moieties and their coordination sphere is a slight modification of the [Ru([9]aneS_3_)(en)Cl]^+^ complex (**12**, Fig. 11, top left, [9]aneS_3_ = 1,4,7-trithiacyclononane, en = ethylenediamine), which was already shown by the same group to be characterized by a strong cytotoxicity.^41,42^

**Fig. 12:**
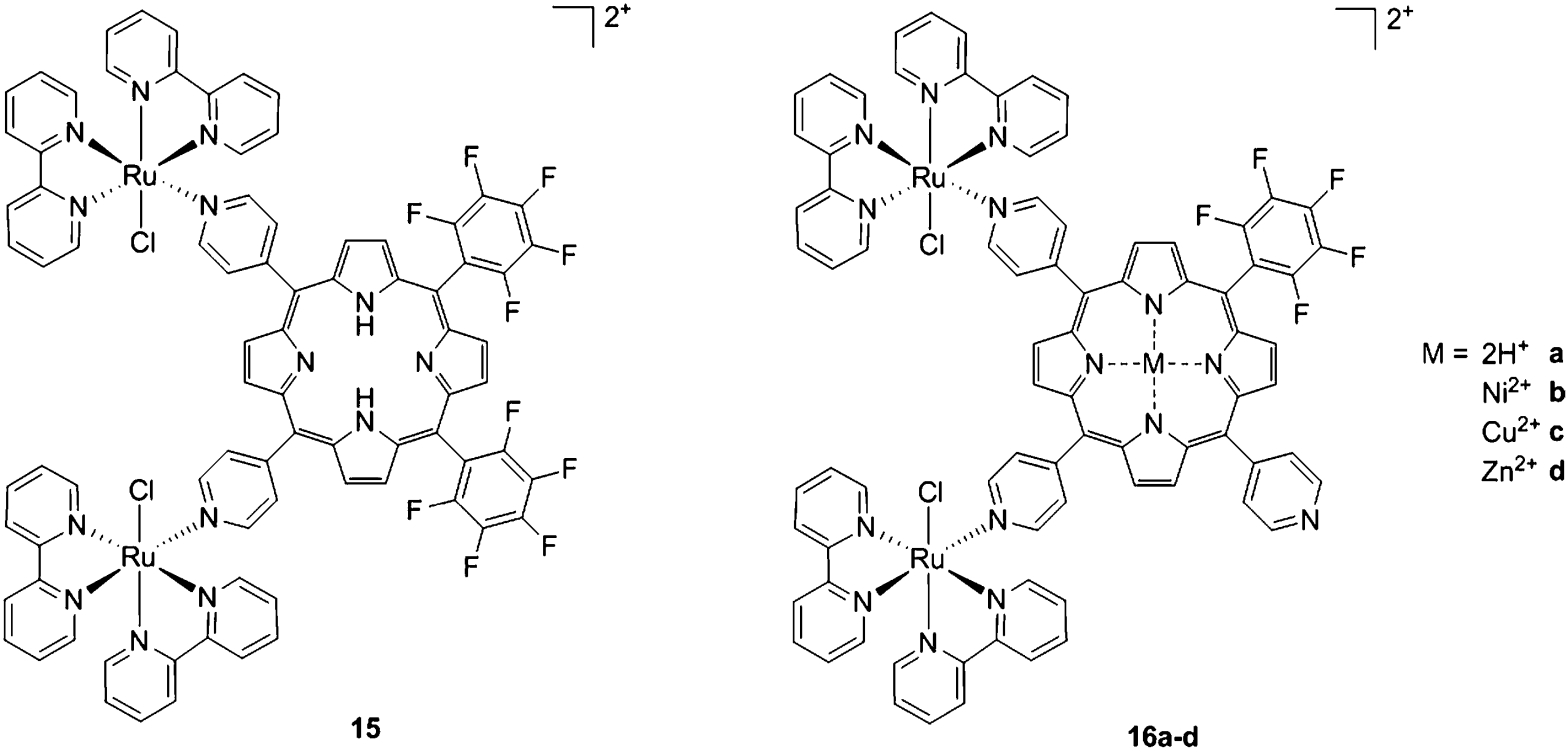
Porphyrin with pentafluoroaryl and Ru(bipy)_2_Cl fragments to give **15** (left) and Ru–porphyrin conjugates containing different metals in the ring (**16a–d**, right).^43,44^

As expected, the ruthenium fragments strongly improved the physicochemical behavior of the porphyrin core. This resulted in a clear increase in cytotoxicity of the compounds, most likely, as speculated by the authors, due to higher cellular accumulation. Furthermore, the potency of the systems in human breast cancer cells MDA-MB-231 was improved by one order of magnitude upon exposure to 5 J cm^–2^ of 590–700 nm light, thus reaching the nanomolar range. Following the same strategy, Swavey *et al.* explored a range of possible modifications of porphyrins to improve their activity and selectivity.^43^ In particular, they introduced a Ru(bipy)_2_ moiety (bipy = 2,2′-bipyridine) with a labile Cl ligand to obtain additional DNA binding and light-induced DNA cleavage. Two pentafluoroaryl groups, which are known to increase the excited state lifetime of a PS, were also linked to the porphyrin, to give compound **15** (Fig. 12, left). The authors obtained a very strong affinity for DNA and consequent photocleavage of plasmid supercoiled DNA. Furthermore, they demonstrated that the compound exerted a higher phototoxicity on melanoma cells when compared to normal skin fibroblast cells.

**Fig. 13:**
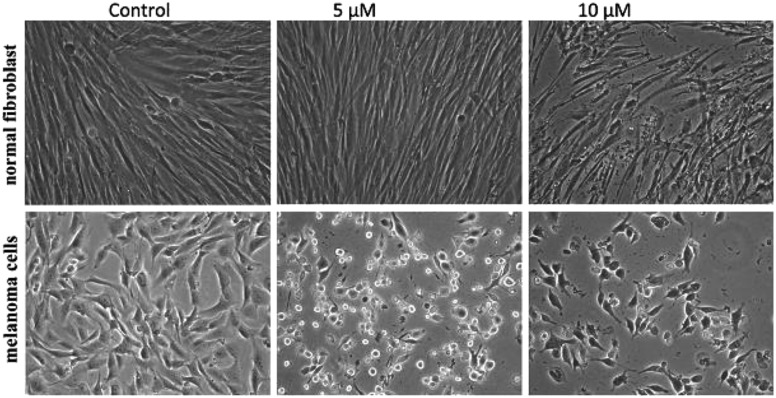
Phase contrast microscopy images of cells irradiated with a 60 W tungsten lamp for 30 min. Normal fibroblast cells (top) and melanoma cells (bottom) without **16d** (control) and in the presence of 5 and 10 μM concentrations of **16d**. Reproduced from ref. 44 with permission from The Royal Society of Chemistry.

To improve the efficacy and the selectivity of their system, the same authors removed one pentafluoroaryl group and evaluated the effect of the insertion of a metal into the porphyrin ring (**16a–d**, Fig. 12, right).^44^ Upon coordination of a metal ion in the porphyrin, the photophysical properties of the system undergo an important change due to the metal perturbing the energy levels of the free ligand. For instance, it was noticed that the complexation of Zn(ii) increases the lifetime of the excited state of the porphyrin.^19^ In this work, they demonstrated that all three metal-coordinated systems were able to nick plasmid DNA upon induction with light, with the Zn(ii) system **16d** also generating double strand breaks. In cellular studies, Ni(ii) and Cu(ii)–porphyrins were inactive as photosensitizers. On the other hand, the Zn(ii) system at a concentration of 5 μM induced cell death very efficiently on a melanoma cell line upon white light irradiation (Fig. 13, bottom). Interestingly, the same treatment did not show any efficacy on normal skin fibroblast cells (Fig. 13, top), providing indications of a very selective system.

Of utmost interest, the authors performed *in vivo* studies with compound **16d** on *Drosophila melanogaster* to assess its general toxicity in the dark as well as biodistribution.^45^ The compound was found to be harmless for the larvae and during their development. Cellular localization studies were also performed by feeding the larvae with the compound. Confocal microscopy revealed that the molecule was able to accumulate in the cytosol, but also in the nuclei at higher concentration. This suggests that the compound is not readily metabolized.

**Fig. 14:**
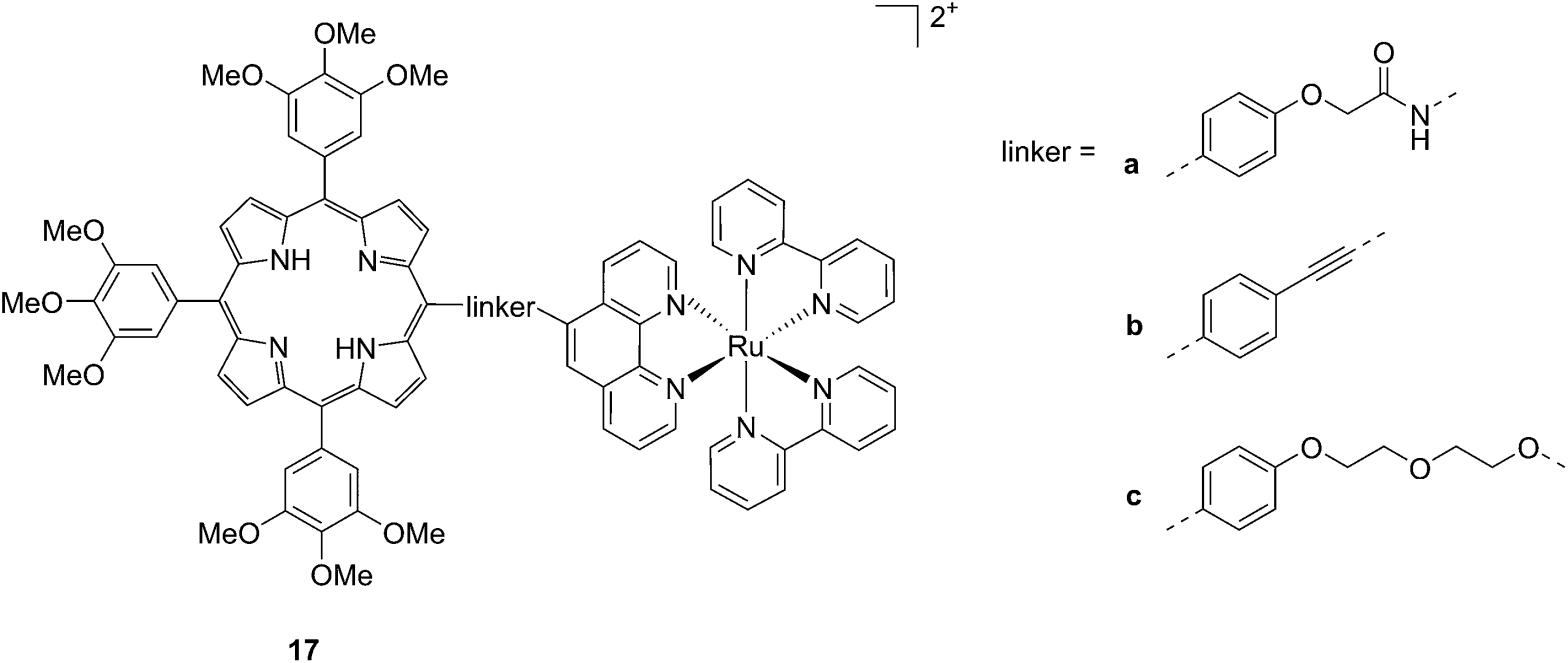
Structures of Ru–porphyrin conjugates **17a–c**, with three different bridging linkers.^52^

Another interesting class of compounds includes the coordinatively saturated ruthenium polypyridyl complexes. These compounds are known to be kinetically inert and substitutionally stable. Therefore, they do not have a labile ligand that can covalently bind DNA. Nevertheless, it was shown that, with the use of appropriate ligands such as dipyrido[3,2-*a*:2′,3′-*c*]phenazine (dppz) or tetrapyrido[3,2-*a*:2′,3′-*c*:3′′,2′′*-h*:2′′′,3′′′*-j*]phenazine (tpph), these complexes can interact very strongly with double-stranded DNA *via* intercalation or groove binding. Thanks to these interesting characteristics, these compounds were extensively studied as DNA intercalating probes^46,47^ or as cytotoxic agents.^48–51^ Furthermore, it was demonstrated that these compounds are also able to produce ^1^O_2_ (see next paragraph for more information on this topic). To exploit this property, Wong and co-workers conjugated a [Ru(bipy)_2_phen]^2+^ (phen = 1,10-phenanthroline) moiety to a porphyrin core *via* three different linkers on the phen (Fig. 14) and evaluated the biochemical behavior of the resulting systems **17a–c**.^52^ The ruthenium conjugation was also introduced here to improve the two-photons absorption (TPA) characteristics of the compounds. As a consequence, by virtue of the simultaneous absorption of two photons, the molecule can be excited at 800 nm, a more tissue penetrating and less harmful wavelength. Therefore, this interesting characteristic allows for the development of bifunctional PDT and tumor imaging agents.

Interestingly, the authors could achieve a different cellular localization based on the type of linker used to connect the porphyrin core to the ruthenium moiety. This difference allowed for studying the effects of PDT in different cellular compartments. Compounds **17a** and **17b** were characterized by the best cellular uptake, as demonstrated by flow cytometry analysis. Comparably, they also displayed the best phototoxic behavior with a toxicity of 118 and 175 μM on HeLa cells in the dark and LD_50_ of 1 μM upon yellow light irradiation with doses of 6.5 and 2.0 J cm^–2^, respectively. Compound **17b** also showed its activity as a TPA-PDT agent, causing cell shrinkage upon irradiation at 850 nm. The compound, which localized in the mitochondria before light exposure, was found to relocate in the nuclei after light irradiation. The authors therefore assumed that **17b** induced light-mediated damage to mitochondria, from which it is then released. Once in the cytosol, the compound can damage the nuclear membrane and cause cell death. Interestingly, they also showed that the presence of the Zn atom in their conjugates had a detrimental effect on the emission quantum yields of the systems in DMSO, going from values of 1.93–5.3% for the free base compounds to <1% when Zn(ii) was inserted in the porphyrin ring. The authors considered this difference in the photophysical behavior to be related to an energy transfer from the Soret band of Zn–porphyrins to the ruthenium fragment.

### Ruthenium complexes as PSs

**Fig. 15:**
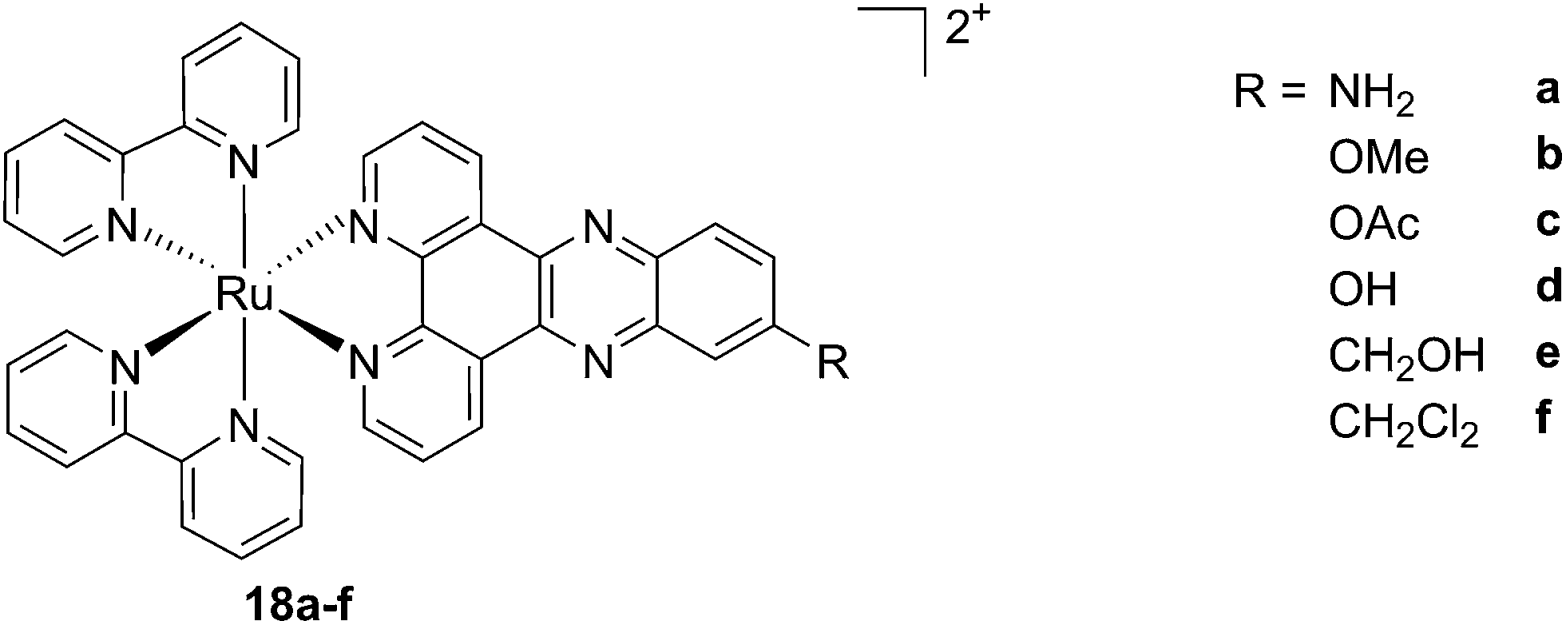
Structures of the six different DNA intercalating Ru complexes **18a–f**.^53^

As discussed above, porphyrins certainly have good characteristics as PSs due to their intrinsic physico-chemical properties. On the other hand, the PSs available on the market still display a number of drawbacks such as their low solubility in biological media, lack of selective cancer accumulation and the frequently encountered photosensitivity in patients undergoing PDT treatments. Over the last few years, several research groups have explored the possibility to move away from tetrapyrrolic systems, studying the potential of metal complexes as PSs themselves. The application of ruthenium complexes as PSs is a reasonable approach due to their tunable photophysics and the aforementioned advantages for biological applications (see Introduction). As an example of this approach, our group synthesized six [Ru(bipy)_2_dppz]^2+^ complexes **18a–f** with different functional groups on the dppz ligand (Fig. 15).^53^

**Fig. 16:**
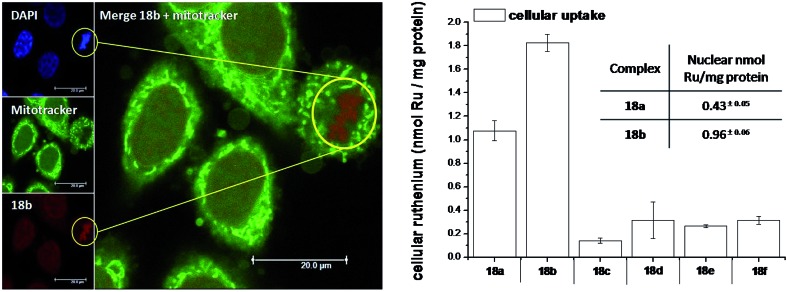
Left: Confocal microscopy images of HeLa cells treated for 2 h with 100 μM of complex **18b** (excitation at 488 nm, emission above 600 nm, bottom left) and stained with DAPI (nuclear staining, top left) and with Mitotracker green (mitochondrial staining, middle left); in the yellow circle a representative example of the different localization of **18b** and Mitotracker green is found (picture on the right). Right: Cellular uptake into HeLa cells treated for 4 h with 20 μM solutions of the complexes **18a–f**. Results are expressed as the mean ± error of independent experiments. In the inset: nuclear uptake for complexes **18a** and **18b**. Reproduced with permission from ref. 53. © 2014 Wiley-VCH Verlag GmbH & Co. KGaA, Weinheim.

As highlighted before, the presence of the dppz intercalative ligand was meant to increase the affinity of the compounds for DNA, so that a targeted delivery of singlet oxygen to the genetic material can be achieved. All Ru complexes were found to be non-toxic (up to 100 μM) to both normal fetal lung fibroblast cells (MRC-5) and cervical cancer HeLa cells in the dark. Nevertheless, the amino- and methoxy-substituted Ru complexes showed impressive photoactivities. When HeLa cells were irradiated with a light dose of 9.27 J cm^–2^ at 420 nm, IC_50_ values in the low micromolar range were obtained for **18a** and **18b**. An impressive PI of 43 for the latter and even >150 for the former were obtained. Cellular distribution studies were performed on both compounds by means of confocal microscopy and high-resolution continuum source atomic absorption spectrometry (HR-CS AAS) and the results are reported in Fig. 16. These techniques indicated a very good cellular uptake of both compounds. Furthermore, HR-CS AAS analysis confirmed the nuclear localization for both complexes after 4 h incubation, allowing for target delivery of ^1^O_2_ to DNA. Compounds **18a** and **18b** also showed good efficiency in generating strand breaks of supercoiled plasmid DNA upon light irradiation. This feature strongly suggested the involvement of DNA in the mechanism of phototoxicity. Further studies are ongoing to investigate the interaction of **18b** with DNA, and the exact mechanism of cell death engendered by light activation.

**Fig. 17:**
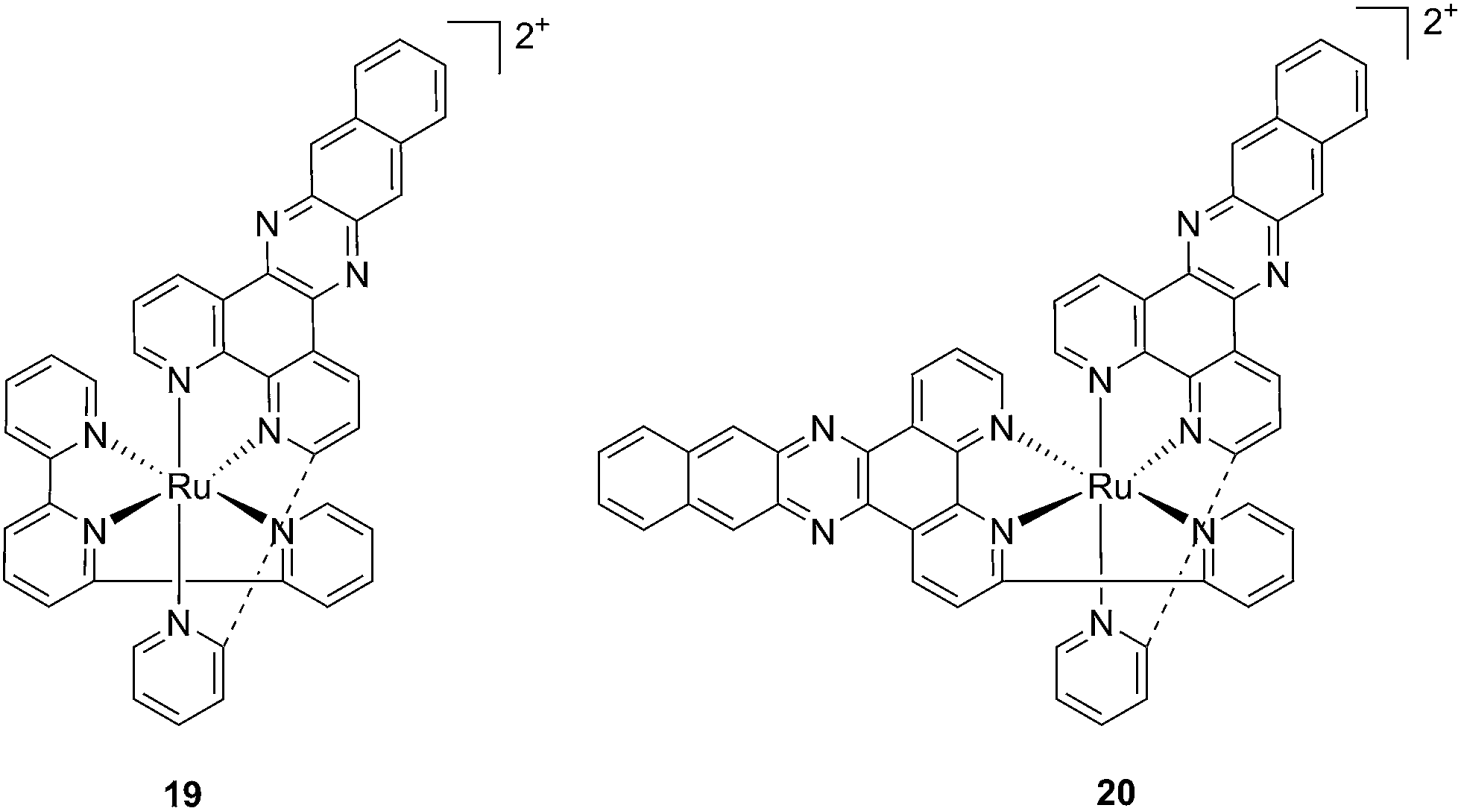
Structures of the Ru complexes **19** and **20** bearing the tridentate pydppn ligand, which confers very long excited state lifetimes.^57^

With the similar goal of targeting and photocleaving DNA, Brewer *et al.* studied mono-metallic or supramolecular complexes of Ru, Pt, Rh and their abilities to interact with DNA upon light irradiation in depth (see also the PACT section below). In particular, they demonstrated the ability of three [(TL)_2_Ru(dpp)]^2+^ compounds (dpp = 2,3-bis(2-pyridyl)pyrazine, with TL = bipy, phen or Ph_2_phen = 4,7-diphenyl-1,10-phenanthroline) to efficiently photocleave supercoiled pUC18 plasmid DNA upon irradiation at *λ* = 450 nm thanks to the formation of ^1^O_2_.^54^ However, the biological activity of compounds of the type [(TL)_2_Ru(dpp)]^2+^ in cells was not evaluated. Turro and coworkers are also very active in the field of light-activated ruthenium complexes. They synthesized and characterized many compounds and studied their photophysics, and light-mediated interactions with DNA and proteins due to the formation of singlet oxygen,^55,56^ or to other mechanisms (see also the PACT section below). To further highlight the mode of action of these photoactivated compounds, these researchers investigated their light-induced effects on DNA and proteins in fibroblasts.^57^ The two complexes, [Ru(tpy)(pydppn)]^2+^ (**19**) and [Ru(pydppn)_2_]^2+^ (**20**) reported in Fig. 17, with tpy = [2,2′;6′,2′′]-terpyridine and pydppn = 3-(pyrid-2′-yl)-4,5,9,16-tetraaza-dibenzo[*a*,*c*]naphthacene,^56^ displayed very long lifetimes of the excited states (20–24 μs), thanks to the pydppn ligand, which allows for singlet oxygen generation with an efficiency of almost 100%.

The authors were then able to demonstrate that **19** and, to a lesser extent, **20** induced photodynamic damage to the tumor suppressor p53 and the DNA polymerase processivity factor PCNA (proliferating cell nuclear antigen), both of them being key components of DNA maintenance and repair pathways. Upon light irradiation of cells and cell lysates (3.15 J cm^–2^ of visible light), the compounds induced covalent crosslinking of the protein subunits, the formation of DNA–protein adducts and, as a consequence, the inhibition of DNA replication. p53 crosslinking was previously demonstrated to correlate with the formation of singlet oxygen,^58^ and the work of Turro and colleagues^57^ demonstrated a strong reduction in the efficiency of p53 photodamage by the presence of sodium azide, a known singlet oxygen quencher. In addition, protein–DNA crosslinking was demonstrated to depend on singlet oxygen-mediated formation of 8-oxo-7,8-dihydroguanine and its further reaction with amino groups in the protein. Also in this case though, the evaluation of the phototoxic profile of the compounds on cells was not explored.

**Fig. 18:**
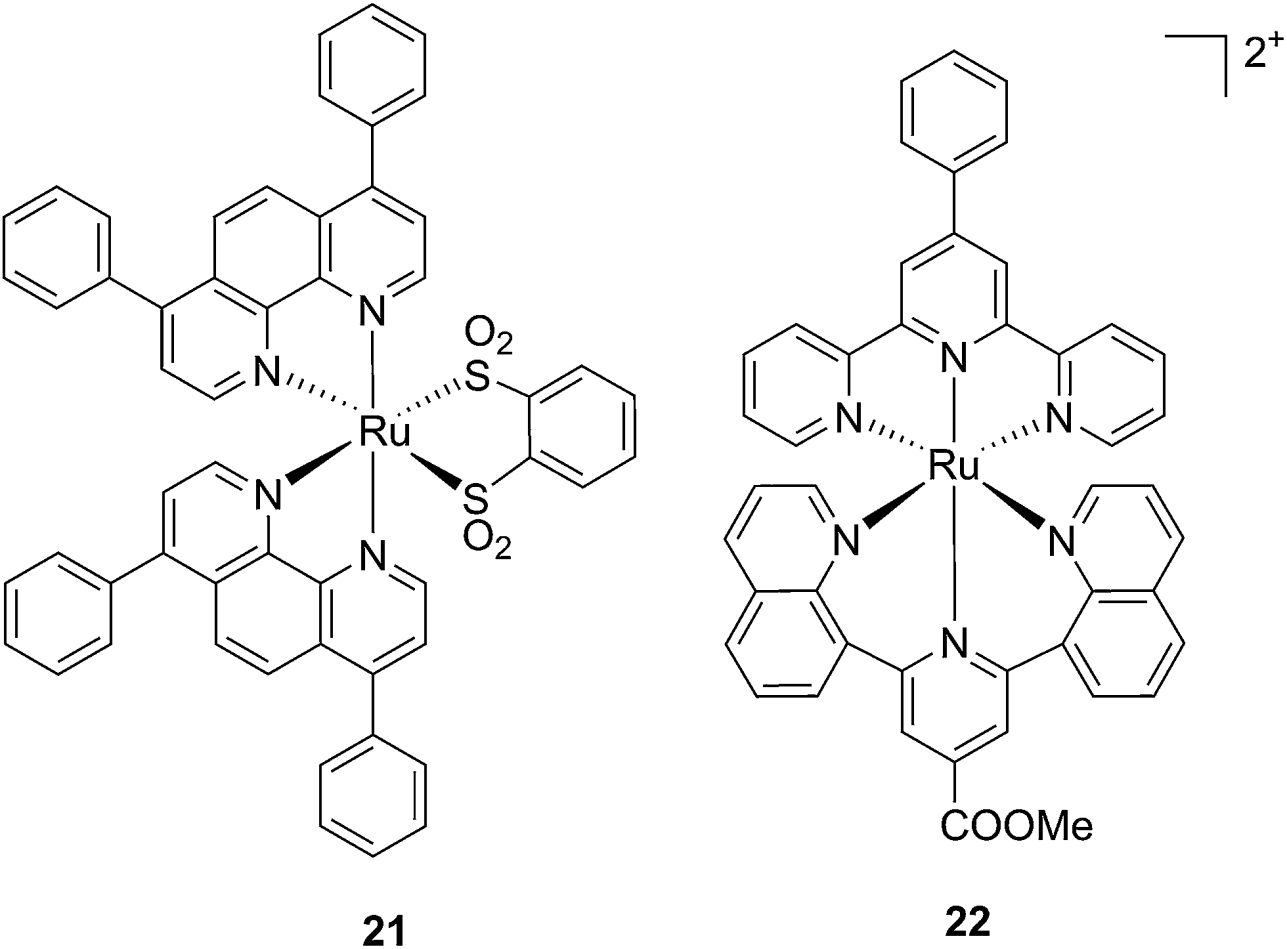
Structures of the ruthenium complexes **21** and **22** which have PDT and aPDT activity.^60^

Ruthenium polypyridyl complexes also have an excellent record of performance in the field of dye-sensitized solar cells (DSSCs)^59^ due to their absorption in the visible range and very long lifetimes. Interestingly, and as previously noted, these characteristics are also of extreme importance in the field of PDT. Consequently, our group decided to explore the photodynamic behavior of two derivatives of ruthenium complexes bearing a benzenedithiol (**21**) and a tridentate polypyridyl ligand (**22**), respectively (Fig. 18), which were previously employed in the field of DSSCs.^60^

**Fig. 19:**
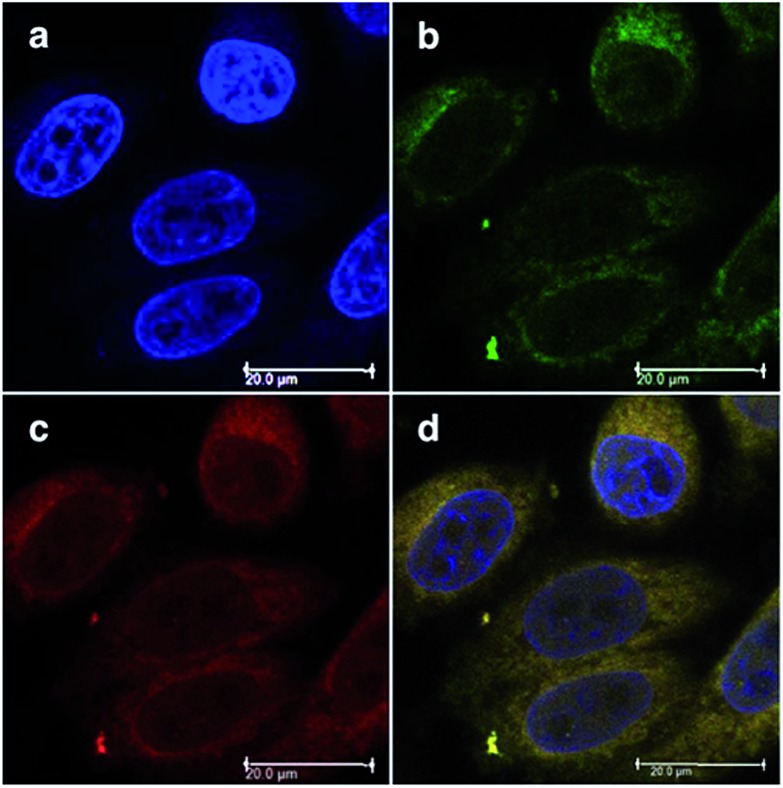
Fluorescence confocal microscopy images of HeLa cells incubated with 40 μM of **21** for 4 h: (a) DAPI staining, (b) Mitotracker green FM staining, (c) visualization of **21** by excitation at 405 nm, (d) overlay of a–c. Reprinted with permission from ref. 60. Copyright 2014 American Chemical Society.

Both compounds were characterized by moderate uptake by HeLa cells, as indicated by inductively coupled plasma mass spectrometry (ICP-MS) analysis performed after 4 h incubation. **21** accumulated preferentially in mitochondria (67% of the entire Ru uptake) as also confirmed by fluorescence confocal microscopy (Fig. 19). **22**, on the other hand, was shown to target the nuclei, where 50% of the total Ru that entered cells was localized.

Phototoxicity was evaluated on HeLa cells. **21** was found to be most active upon irradiation at 420 nm with 6.95 J cm^–2^. Its PI was equal to 80, with an IC_50_ of 620 nM upon light irradiation. It is important to notice that although the uptake of **21** was not as high as those reported for similar complexes, the amount of compound present in cells was sufficient to produce a strong phototoxic effect. On the contrary, **22** displayed a lower phototoxicity against HeLa with an IC_50_ of 25.3 μM under the same irradiation conditions. Of utmost interest, the compounds were also evaluated for their potential activity as PSs in antibacterial PDT (aPDT). The use of PDT to kill bacteria was recently exploited to overcome the problematic occurrence of resistance to available antibiotics. This is essentially due to the fact that a resistance mechanism is far more difficult to develop for bacteria since PDT does not have a specific target but can affect the entire cell. The antibacterial activities of **21** and **22** were tested on the Gram-(–) *Staphylococcus aureus* and on the Gram-(+) *Escherichia coli*. Surprisingly, **22** was active against both strains, with a reduction of >6 log_10_ of the viability of the *S. aureus* and >4 log_10_ of that of *E. coli* at a concentration of 50 μM and with a dose of 8 J cm^–2^ of light at 420 nm. Under the same conditions, **21** displayed the same activity towards *S. aureus*, while being completely non-toxic towards *E. coli*. The very good performance of **22** is particularly promising considering that it is reported that normally Gram-(–) bacteria are less sensitive to PDT treatment.

**Fig. 20:**
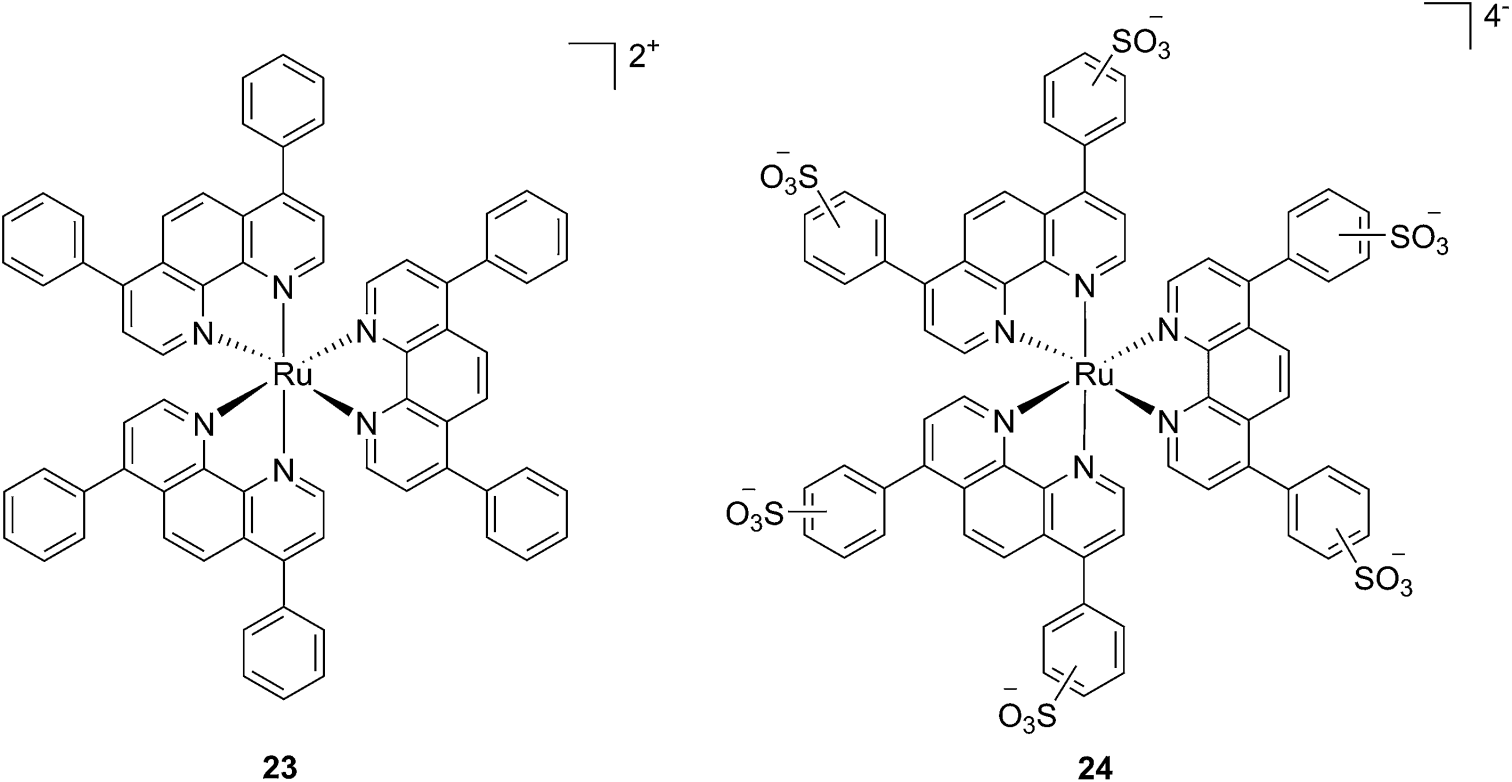
Structures of the Ph_2_phen complexes **23** and **24** with different charges investigated by Glazer and co-workers.^61^

In the last few years, Glazer and coworkers have thoroughly investigated the application of Ru polypyridyl complexes as PACT agents (see also PACT section). However, they also recently performed an in-depth biological characterization of two potential PDT agents. In particular, they evaluated [Ru(Ph_2_phen)_3_]^2+^ (**23**) and [Ru(Ph_2_phen-SO_3_)_3_]^4–^ (**24**) (Fig. 20), which are known dyes for solar cells or biological staining, but which were never investigated as PDT agents.^61^ The two compounds have very similar structures but extremely different physical properties, mainly due to their different charges, namely +2 for **23** and –4 for **24**. This, along with the subsequent difference in hydrophilicity of the two molecules, was expected to induce distinct cellular responses. Nonetheless, both molecules were found to be able to produce singlet oxygen when photo-irradiated.

**Fig. 21:**
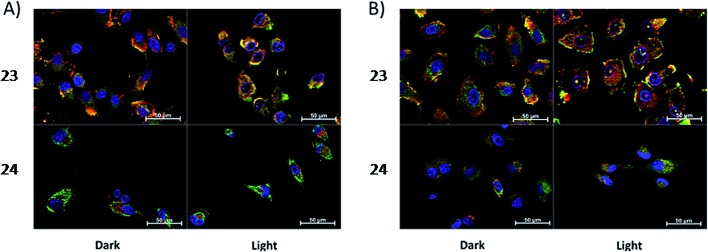
ApoTome microscopy showing subcellular localization of **23** and **24** at 8 h. Co-localization of **23** and **24** in mitochondria or lysosomes is indicated by the apparent yellow emission. (A) Mitotracker green FM was used to image mitochondria. (B) Lysotracker green DND-26 was used to image lysosomes. Red color denotes intrinsic emission of **23** and **24**, whereas blue color denotes Hoechst staining of the nucleus. The yellow color results from overlap of the red emission from the ruthenium complexes and green emission of the organelle-specific dyes, indicating co-localization. Compound **23** localizes in both the mitochondria and the lysosomes, while **24** was not predominantly found in either organelle. Reprinted with permission from ref. 61. Copyright 2014 American Chemical Society.

Toxicity experiments were performed on three different cell lines (A549 human non-small lung cancer cells, HL60 human promyelocytic leukemia cells and Jurkat human T lymphoblastoid cells) in the dark and upon irradiation with 7 J cm^–2^ of >400 nm light. **23** showed a very good cytotoxic effect on all cell lines studied. Irradiation brought a further increment in potency, with IC_50_ values ranging from 0.075 μM to 0.35 μM, depending on the cell lines employed. However, the PI was just around 10–20. Surprisingly, **24** appeared to be non-toxic in the dark (up to 300 μM) on all cell lines studied. Nevertheless, irradiation induced strong toxicity with IC_50_ values in the low micromolar range, resulting in a larger therapeutic window compared to **23**. The compounds also displayed a different subcellular localization, with **23** accumulating in mitochondria and lysosomes and **24** displaying a non-specific accumulation in the cytoplasm (Fig. 21). Interestingly, mitochondrial uptake of **23** was proposed by the authors as the cause of toxicity in the dark. Upon light irradiation, **23** relocalized from mitochondria and lysosomes to the nucleus. This phenomenon was explained by the authors as the consequence of damage to the nuclear membrane induced by **23** upon light irradiation. On the other hand, when cells incubated with **24** were irradiated, the compound was mainly observed in lysosomes, suggesting that no damage occurred to the nuclear membrane in this case.

Investigation of the mechanism of cell death using distinct assays and read-outs revealed a role for light-induced apoptotic pathways in the case of **24**. On the other hand, initial necrotic cell death in the dark, followed by a combination of necrotic and apoptotic pathways, was observed for **23** upon light irradiation.

**Fig. 22:**
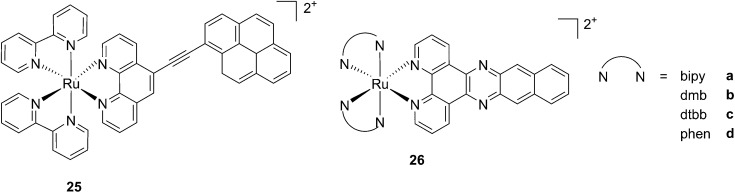
Structures of ruthenium complexes **25** and **26** studied by McFarland, characterized by ^3^IL excited states.^62,64^

While one of the main problems of PDT is its reliance on oxygen, which is often present at low concentrations in the tumor environment (hypoxic conditions), the application of metal complexes as PSs also has its drawbacks, which are due to the need for light at a high energy (blue or green) for the excitation of the PS. McFarland and co-workers addressed both issues by taking advantage of the possibility to fine tune the photophysical characteristics of coordination compounds. More specifically, by modifying the structures of the ligands coordinated to the metal centre, the authors developed Ru polypyridyl PSs characterized by a triplet intraligand (^3^IL) excited state with remarkably long lifetimes. Oxygen was reported to be able to quench this excited state even when present at very low concentrations (3.5%). Furthermore, the strong photosensitizing ability of this excited state allowed PDT effects to be achieved in the red and NIR regions where compounds have marginal absorptions (*ε* values in the order of 10 M^–1^ cm^–1^). The first series of compounds bearing a pyrenylethynyl moiety on the phenathroline ligand was strongly effective on the cell line Malme-3M, a malignant melanoma lung metastasis.^62^ Melanoma cells are able to grow at very low oxygen concentrations and have a remarkable ability to resist the outburst of ROS.^63^ Nevertheless, compound **25** (Fig. 22, left) could induce cell death in a melanoma cell line, with a toxicity increase of two orders of magnitude upon irradiation with white light at 7 J cm^–2^. In these conditions, EC_50_ went from 62 μM in the dark to 200 nM upon irradiation.

**Fig. 23:**
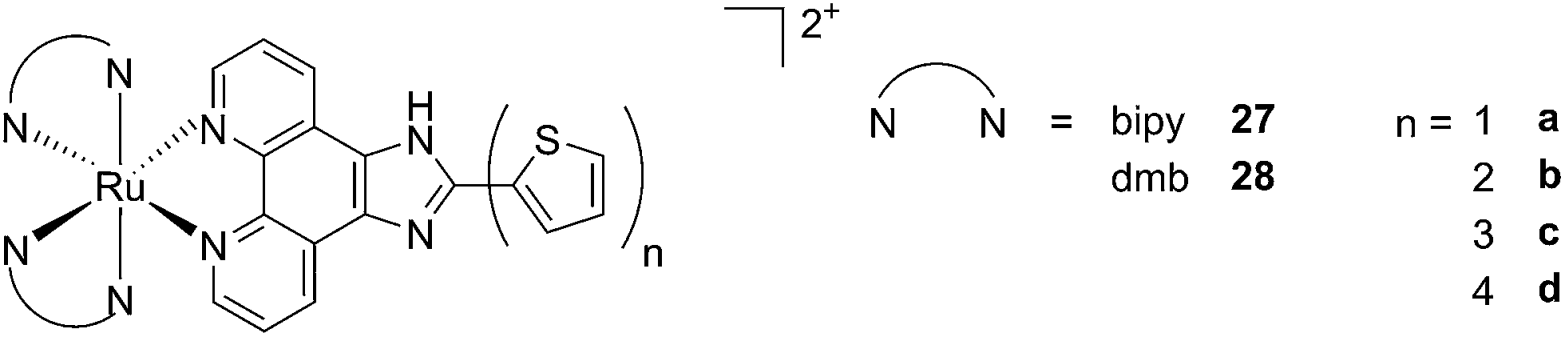
Structures of Ru polypyridyl complexes conjugated with different polythiophene moieties to achieve dual Type I/II photosensitization.^65^

A second class of compounds studied by the same group contained the extensively conjugated benzo[*i*]dipyrido[3,2-*a*:2′,3′-*c*]phenazine ligand (dppn, Fig. 22, right).^64^ The authors could exploit the ^3^IL excited state of these compounds with very long lifetimes to obtain a remarkable PDT effect. Impressively, EC_50_ values in the low micromolar range were obtained upon irradiation with 100 J cm^–2^ light at 625 nm, where the compounds have marginal absorption. This efficacy demonstrated that it is possible to achieve good photoactivity with compounds that mainly absorb in the blue-green region of the light spectrum. Furthermore, the same authors developed a system where the Ru polypyridyl complexes are connected to polythiophene chains of variable lengths (Fig. 23). This conjugation gave access to a low-lying ^3^IL excited state and to a strong non covalent DNA association.^65^ Gel electrophoresis experiments were performed on the complexes to elucidate the interaction with plasmid DNA. These analyses suggested that compounds bearing more than one thiophene unit are able to induce light-mediated damages to plasmid DNA *via* an oxygen-independent pathway. This was indicated by the fact that compound **27c** was still able to induce single strand breaks when the experiment was performed under argon atmosphere. Therefore, the authors speculated that these thiophene conjugates could act *via* photoinitiated Type II reaction in the case of high oxygen tension. On the contrary, under low oxygen concentration, the compounds induced damage to DNA *via* a Type I pathway. This behavior was already observed for this class of compounds in the photoinactivation of bacteria.^66^ A PS with the ability to act *via* a dual Type I/II photosensitization could allow for the treatment of hypoxic tissues, broadening the spectra of applicability of PDT.

The *in vitro* PDT effect of these compounds was found to be directly proportional to the polythiophene chain length, with a PI of > 200 when 4 thiophene units were present in the complex. The two best compounds **27c** and **28c**, bearing three thiophene units, were also tested *in vivo* on mice, which were inoculated with colon carcinoma cells (CT26.WT). In animals treated with compound **28c** (53 mg kg^–1^), administration of 525 nm continuous wave light (192 J cm^–2^) resulted in complete tumor regression, with no recurrence up to 52 days after the end of the treatment. These compounds are currently under optimization for clinical phase I trials.^65^

**Fig. 24:**
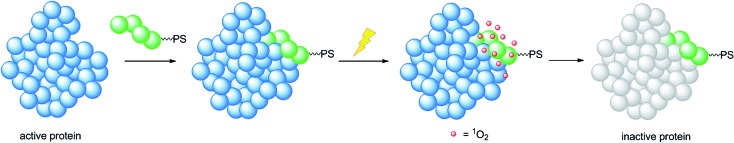
Mechanism of the CALI strategy to inhibit enzymes, applied by the Kodadek group.

A very elegant approach to effectively increase the selectivity of PDT treatment is the so-called CALI (chromophore-assisted light inactivation). This technique is based on the functionalization of a modest protein inhibitor with a PS, allowing for strong enhancement of the inhibitory properties through photo-triggered ^1^O_2_ generation in close proximity to the active site (see the mechanism in Fig. 24). The Kodadek group recently explored this technique using a [Ru(bipy)_3_]^2+^ derivative,^67^ demonstrating the feasibility of this technique on both membrane and intracellular proteins.

Furthermore, the authors showed selective inhibition of RBBP9 serine hydrolase, which is implicated in pancreatic cancer, in protein-enriched cell lysate.^68^ The limitation of this approach is related to the choice of PS, since [Ru(bipy)_3_]^2+^ derivatives do not allow efficient photosensitization due to the short wavelengths required for excitation. A careful optimization of the system could provide a very useful tool for future targeted PDT applications.

## Ruthenium complexes in PACT

As mentioned above, PDT relies on the presence of oxygen to induce cell death. However, most tumors are hypoxic in their internal core,^69^ limiting the efficacy of PDT. Hence, increasing efforts are devoted to the optimization of novel photo-activation strategies that do not rely on an oxygen-dependent mechanism, but which would still allow for spatial and temporal control of the toxicity engendered to cells. Strategies of this type are normally referred to as photoactivated chemotherapy. In this section of the review, we describe the recent efforts in the use of ruthenium complexes for PACT. We have divided this section into two main parts depending on the photo-activation strategy employed. In the first part, we will focus on ruthenium-based DNA photobinders acting (1) in a cisplatin-like mode of action resulting in DNA helix distortion; (2) *via* intercalation yielding DNA cleavage; and (3) *via* conjugated oligodeoxyribonucleotides (ODNs) to allow for gene silencing. In the second part, we will discuss photo-activated release approaches involving Ru(ii) complexes. In this part, we will first introduce the use of Ru(ii) complexes as caging agents for the selective release of bioactive molecules upon light activation. We will then present a parallel approach consisting of the photorelease of cytotoxic Ru(ii) complexes rendered inactive upon caging.

### Photo-activated Ru complexes targeting DNA

Cancer cells differ from their original healthy precursor cells by their ability, *inter alia*, to continuously proliferate.^70^ This feature, conferred by mutations in tumor suppressor genes or by the altered expression/activity of proto-oncogenes, implies continuous activation of DNA replication, which is not the case in healthy cells, which rather display the ability to enter quiescence after a certain number of cell divisions. This hallmark of cancer has been extensively exploited to selectively target cancer cells by means of chemotherapeutic drugs, inhibiting components of the DNA replication/transcription machineries, such as topoisomerase I (*e.g.* camptothecin)^71^ or covalently binding to DNA (*e.g.* cisplatin)^72^. In this section, we will introduce photo-triggered strategies designed to target DNA.

#### Ligand photo-dissociation and DNA target

The best known metal complex used in cancer treatment is undoubtedly cisplatin, a metal-based drug that targets growing cells by interfering with DNA replication. Cisplatin is a prodrug that first undergoes a process called aquation, by which chloride ligands are displaced by water. The cytotoxic activity of cisplatin results from interaction of the highly reactive hydrated form of the drug with DNA, preferentially with the N7 atoms of purine residues.^73^ The majority of lesions generated by cisplatin consist of intra-strand cross-links at two consecutive purines that are promptly addressed by the Nucleotide Excision Repair pathway. On the other hand, the far smaller proportion of inter-strand crosslinks causes distortions of the double helix and inhibits replication,^74^ transcription^74,75^ and translation,^76^ representing a serious threat to cell survival. Furthermore, although replication fork stalling at inter-strand crosslinks does not compromise the completion of S-phase, as it is compensated for by incoming forks from the opposite site of the lesion, the real threat consists of the persistence of the inter-strand crosslink until mitosis, leading to apoptosis.^77^ The broad spectrum of action of cisplatin, as well as its lack of specificity for cancer cells, is evidenced by the severe side effects observed in patients treated with the drug (*e.g.* nephrotoxicity, ototoxicity, *etc.*).^72^ Hence, a significant effort has been directed towards more targeted strategies, involving the use of an external trigger such as light to induce cytotoxicity. As an example, Sadler and coworkers have designed and characterized photoactivatable cisplatin derivatives with clear potential for use in PACT.^78–80^ In particular, they showed that irradiation of Pt(iv) diazido derivatives with UV-A or blue light induced photoejection of the azido ligands and reduction of the metal to Pt(ii). As a consequence, the photoproduct can covalently bind DNA in a similar way to cisplatin, generating a potent cytotoxic effect on cells in culture.^20,81^

**Fig. 25:**
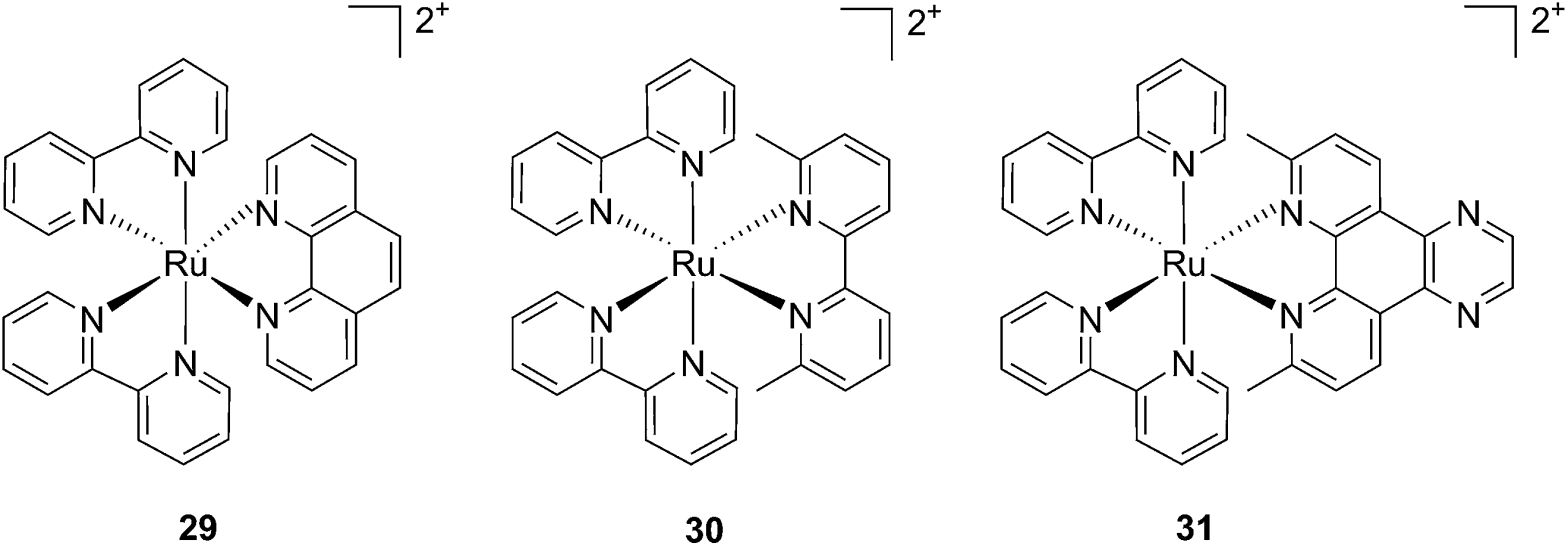
Structures of the strained Ru complexes **30** and **31** that undergo ligand photoejection and the inert control **29**.^84^

In contrast to square planar Pt(ii) compounds, Ru(ii) complexes offer octahedral conformations. It was shown that complexes with distorted octahedral geometry can undergo ligand dissociation after photo-irradiation,^82,83^ which is followed by the formation of an aqua complex that can bind to DNA in a manner similar to cisplatin. To exploit this concept, Glazer and coworkers recently investigated the potential use of methyl substituents on one polypyridyl ligand to obtain highly distorted geometries.^84^ To this end, an unstrained [Ru(bipy)_2_phen]^2+^ (**29**) and two methylated derivatives of [Ru(bipy)_2_(2,2′-bypiridyl)]^2+^ (**30**) and [Ru(bipy)_2_(dipirido[3,2-*f*:2′,3′-*h*]-quinoxaline)]^2+^ (**31**) were synthesized (see Fig. 25 for structures). As expected, after >450 nm light irradiation using a 200 W projector, the authors could monitor the photoejection of the methylated ligand of **30** and **31**, with half-lives (*t*_1/2_) of 2 and 60 min, respectively. Since photoejection of the latter ligand resulted in the formation of a similar aqua species to cisplatin, the authors naturally explored the activity of the photo-product on biologically relevant molecules. In the presence of plasmid DNA pUC19, only irradiated (>450 nm, 200 W, 1 h) products showed DNA damage. Complex **29** produced DNA photo-cleavage, **30** showed only DNA photobinding whilst **31** combined both properties. To verify if the DNA damage observed *in vitro* would reflect in decreased viability in cancer cells in culture, the authors treated HL60 leukemia and A549 lung cancer cells for 12 h with the complexes in the dark prior to >450 nm irradiation for 3 min at 410 W, followed by a further 72 h of incubation (see Table 1 for complete IC_50_ values). As a control, they used aminolevulinic acid (ALA) which is a clinically available PS. Complexes **30** and **31** showed no toxicity in the dark with IC_50_ values of > 100 μM. However, a strong effect after irradiation on both cells with IC_50_ on HL60 cells of 1.6 and 2.6 μM, respectively, and of 1.1 and 1.2 μM, respectively, on A549 cells, was observed. In order to efficiently mimic the three-dimensional tumor environment, the authors also assessed the photo-toxicity of the compounds on A549 spheroids. As reported for the monolayer cell culture, complex **30** was confirmed to be efficient with an IC_50_ of 21 μM upon light irradiation, a value that corresponds to twice the potency of cisplatin on the same spheroids. Worthy of note, glutathione (GSH), responsible for cisplatin inhibition in cells, had no deleterious effect on DNA binding or cleavage efficiency nor on the toxicity of the ruthenium complexes **30** and **31**.

**Table 1:** Cytotoxicity IC_50_ values in 2D and 3D cellular assays^*a*^

IC_50_ (μM)								
Compounds	Light			Dark			PI	
	HL60	A549	A549 spheroid	HL60	A549	A549 spheroid	HL60	A549
Cisplatin	3.1 ± 0.2	3.4 ± 0.6	n. d.	3.1 ±0.1	3.5 ± 0.6	42 ± 3.6	1	1
**29**	8.1 ± 1.9	40 ± 4	>300	240 ± 9	250 ± 5	>300	3	6.3
**30**	1.6 ± 0.2	1.1 ± 0.3	21.3 ± 2.3	>300	150 ± 7	>300	>188	136
**31**	2.6 ± 1.0	1.2 ± 0.1	64.6 ± 4.7	108 ± 1.9	250 ± 5	>300	42	208
ALA	16.2 ± 3.2	21 ± 3.5	>300	>300	87.8 ± 5.5	>300	>18	4.2

^*a*^n. d. = not determined. ALA = aminolevulinic acid.

**Fig. 26:**
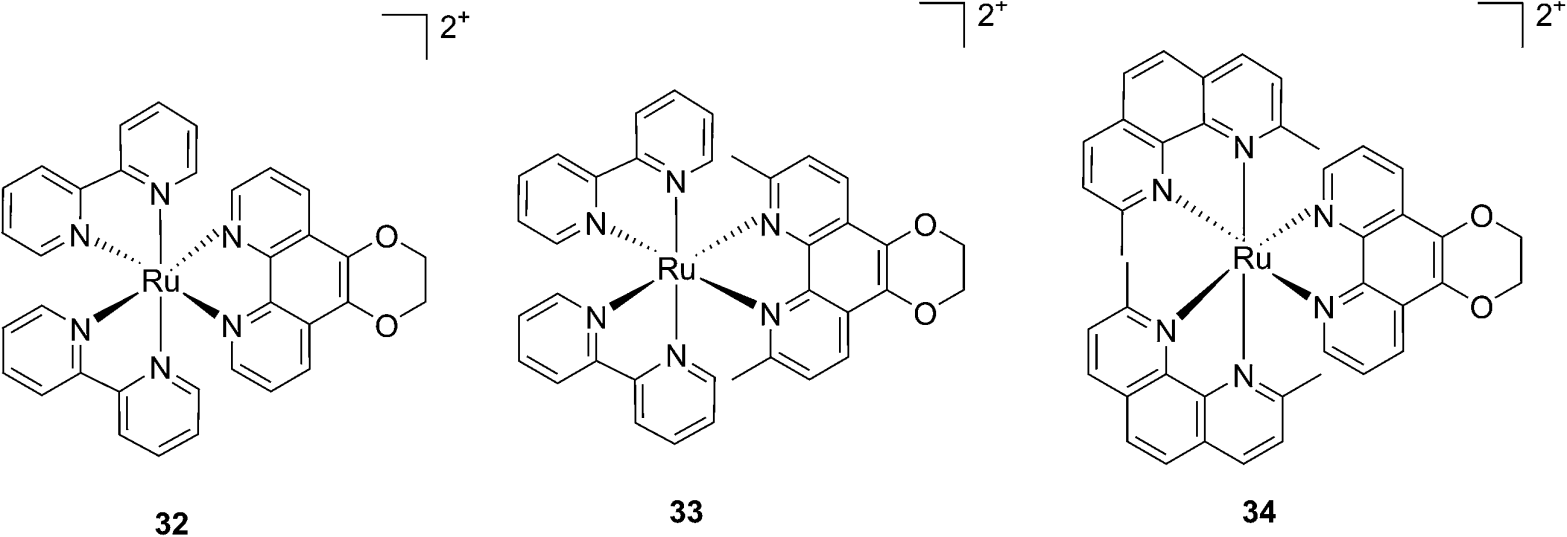
Structures of the photostable control compound **32** and the strained **33** and **34** that undergo ligand photoejection.^85^

More recently, Glazer *et al.* applied the same methylation strategy to a novel strained Ru(bipy)_2_ complex bearing a 2,3-dihydro-1,4-dioxino[2,3-*f*]-1,10-phenanthroline (dop) (**32**) ligand.^85^ The methylated form of **32** is 2,3-dihydro-1,4-dioxino[2,3-*f*]-2,9-dimethyl-1,10-phenanthroline (dmdop) (**33**, Fig. 26). To further increase the straining, the authors also synthesized a [Ru(dmphen)_2_(dop)]^2+^ (dmphen = 2,9-dimethyl-1,10-phenanthroline) (**34**, Fig. 26). As anticipated by the authors, after irradiation at >400 nm with a 200 W projector at a distance of 12 inches from the cuvette, both methylated analogs showed photoejection. The process was found to be 10-fold faster for complex **34** (*t*_1/2_ = 4 min) than for **33** (*t*_1/2_ = 42 min). The authors further analyzed photo-induced DNA damage. Upon the same irradiation settings, complex **32** created single strand breaks (SSBs) in pUC19, likely *via* ^1^O_2_ production. In comparison, complex **34** showed covalent binding while **33** showed a combination of both mechanisms. Regarding cell photo-toxicity, complex **34** exerted the highest toxicity against leukemia cells HL60 with a PI of >1880. The IC_50_ was 300 μM in the dark while the value was 0.16 μM after 12 h incubation, 3 minutes irradiation at >400 nm with 410 W projector and 72 h recovery. This impressive PI was explained by the fact that **34** binds and distorts DNA, whereas the mechanism of action of complex **33** is characterized by a dual mode of action including SSBs formation *via* ^1^O_2_ production and DNA distortion, which possibly lowers its distortion efficiency.

**Fig. 27:**
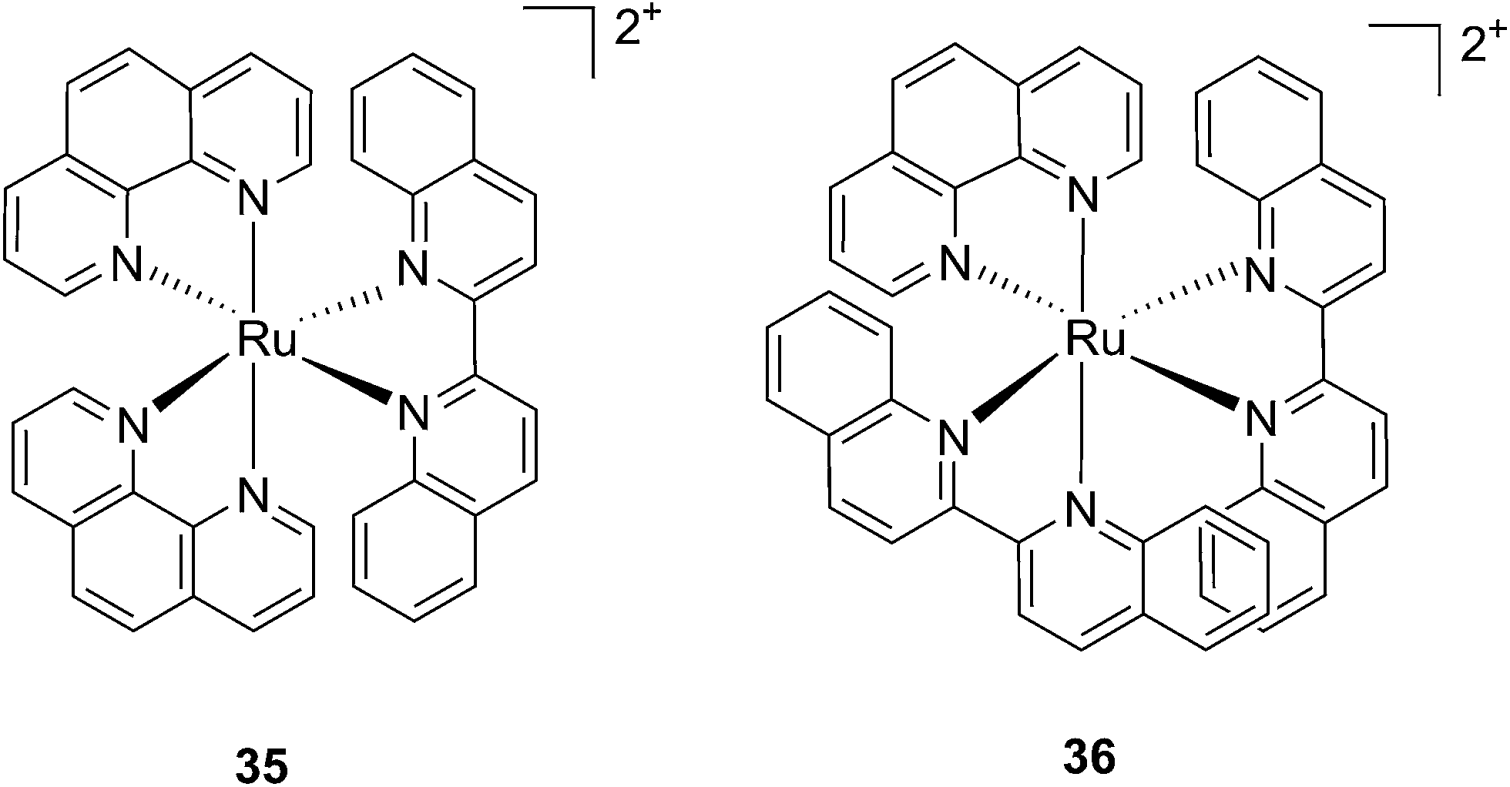
Structures of the two Ru(ii) complexes **35** and **36** with the biq ligands that are exchanged upon irradiation.^86^

Following a similar distortion strategy, the same group studied the coordination of biquinoline (biq) to ruthenium-based complexes. Such a ligand would act as a potent geometry distorter, and at the same time, improve the light activation process by pushing the absorption maximum to higher wavelengths. Indeed, the resulting strained Ru(ii) 2,2′-biquinoline complexes (see Fig. 27 for structures) were shown to be active in the PDT therapeutic window. **35**, which bears one biq, and **36**, which contains two biq ligands, can absorb light up to 700 and 800 nm, respectively.^86^ Both complexes induced decreased electrophoretic migration of the DNA plasmid pUC19 only upon illumination (samples were placed at 12 inches from a 200 W lamp equipped with either blue, green, red or near-IR cut-off filters and irradiated for 1 h or 3 h). Since the appearance of open circular or linear DNA was not observed, the mechanism involved clearly coincides with photobinding. Maximal activity has been observed with blue light irradiation, which is consistent with the absorption profile of the Ru(ii) complex. Cytotoxicity against HL60 leukemia cells followed the same light-dose/-wavelength profile as DNA photo-cleavage. After 12 h incubation followed by 7 J cm^–2^ light irradiation and 72 h recovery, a cell viability assay revealed that complex **36** had the best photo-toxicity profile with IC_50_ values of between 2.3 and 5.1 μM among the different wavelengths used, compared to 47.3 μM in the dark (see Table 2 for IC_50_ values). The interesting PIs (blue: 19.7, IR: 9.2) obtained for complex **36** hold great promise for this type of Ru(ii) complex, which can be activated with red light or even with near-IR wavelengths. Interestingly, a complex similar to compound **36** but bearing a 2-phenylpyridine (phpy) instead of the phen, had different photophysical properties. Indeed, Dunbar, Turro and coworkers found that the latter complex had enhanced phototoxicity against HeLa cells.^87^ Nevertheless, its mode of action remains unclear, since no ligand dissociation was observed. Moreover, the short lifetime of the compound rules out any singlet oxygen mechanism.^88^

**Table 2:** Photobiological activities of **35** and **36** on HL-60 cells^*a*^

IC_50_ (μM)						PI	
Compounds	Dark	Blue (3 min)	Red (3 min)	Red (6 min)	IR (25 min)	Blue	IR
**35**	52.5	1.2	13.8	7.6	15.8	43.8	3.32
**36**	47.3	2.4	4.5	2.3	5.1	19.2	9.2
Cisplatin	3.1	3.1	n. d.	n. d.	n. d.	1	

^*a*^n. d.= not determined.

**Fig. 28:**
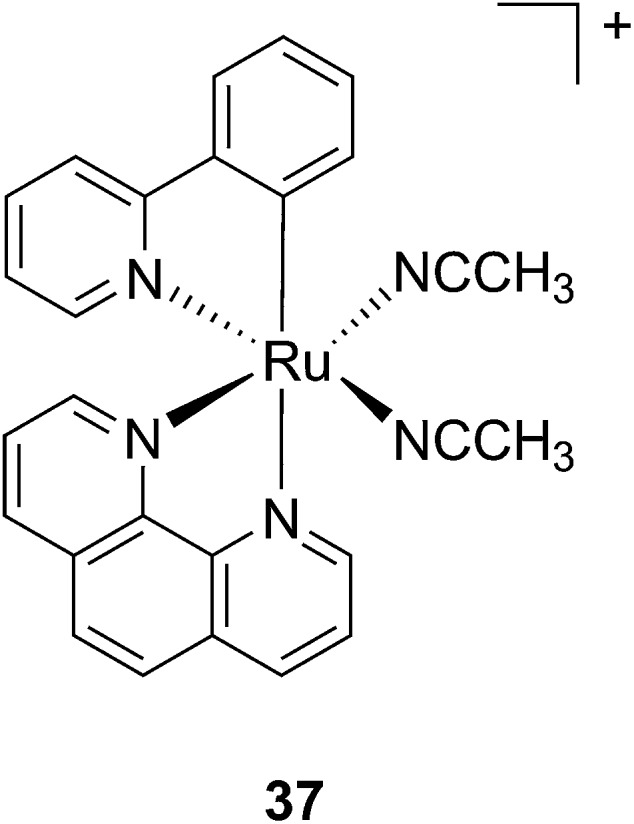
Structure of the Ru(ii) complex **37** with labile CH_3_CN ligand.^91^

Another manner to shift the MLCT absorption of a Ru(ii) complex to the red (PDT window) is by insertion of a cyclometallating ligand.^89^ For this purpose, Turro and coworkers focused on the ligand phpy. More specifically, they investigated the photo-induced ligand release of *cis*-[Ru(phpy)(phen)(CH_3_CN)_2_]^2+^ (**37**, Fig. 28), a complex known to decrease tumor growth in mice,^90^ but whose photo-induced ligand release potential had never been evaluated. First, they observed that 3 min of irradiation at 690 nm were sufficient to eject one CH_3_CN, while 30 min were needed to release the second acetonitrile ligand. They could also observe an enhancement of cytotoxicity upon light irradiation (100 s irradiation at 690 nm, 5 J cm^–2^). As expected, the compound displayed a potent cytotoxicity on human advanced ovarian epithelial cancer cells (OVCAR-5) in the dark, with an IC_50_ of 1 μM (15 h incubation followed by rinsing and then by 24 h recovery). Upon light irradiation, the IC_50_ reached 70 nM, a 14-fold increase compared to dark conditions. According to agarose gel shift assay, this increase in toxicity upon light illumination is due to photobinding of the complex to DNA. Of note, the authors could demonstrate that GSH enhanced the photo-dissociation process, but still further analyses are needed to fully understand its role.^91^

**Fig. 29:**
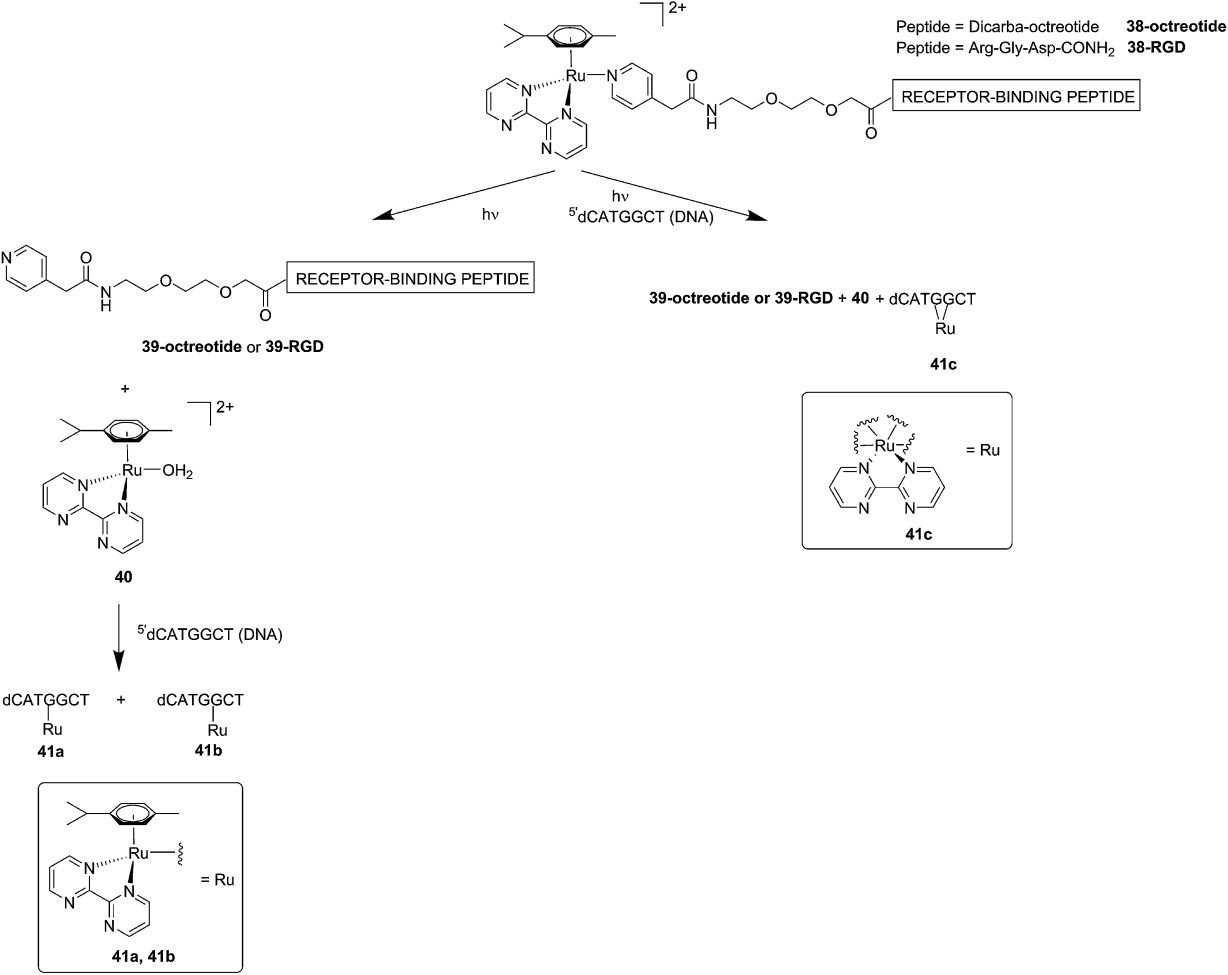
Schematic representation of the photo-dissociation process. Left branch: pre-irradiated Ru–peptide conjugates followed by addition of the oligonucleotide. Right branch: irradiation of a mixture of peptide-conjugated Ru(ii) complex and the oligonucleotide.^94^

Although light-activated prodrugs offer an already high temporal and spatial selectivity *per se*, they still suffer from the recurrent problem of photosensitivity. Despite the fact that Ru complexes may be mimicking Fe uptake and thereby accumulate in cancer cells overexpressing transferrin,^92,93^ and assuming an intravenous administration, the light-activatable prodrugs presented above are supposed to be transported everywhere in the body without special selectivity for cancer cells. This implies that surrounding healthy tissues are not free from deleterious effects. To tackle this important drawback, several groups have envisaged coupling a targeting moiety to light-activatable prodrugs. In this perspective, Marchán *et al.* coupled two different receptor-binding peptides, which are known to target receptors overexpressed on the membrane of some cancer cells, to a photoactivated Ru(ii) arene complex (Fig. 29).^94^ Since tumor endothelial cells overexpress two types of integrins, α_ν_β_3_ and α_ν_β_5_, the authors attached the specific targeting peptide sequence Arg-Gly-Asp (RGD). On the other hand, they decided to target the somatostatin receptor sst_2_, which is located at the membranes of malignant cells in supernumerary copies, using the peptide octreotide, a somatostatin agonist. The authors reported interesting and promising *in vitro* studies. First, they showed that conjugation to the peptides did not affect the photo-activation process, since the reactive aqua species was formed after pyridine ligand loss upon visible light irradiation (420 nm lamps). Second, they reported that DNA binding of the Ru-conjugates was not compromised by the presence of the peptides. When pre-irradiated (8 h) peptide-conjugated Ru(ii) complexes **38-octreotide** or **38-RGD** were incubated overnight with the 9-ethylguanine, monofunctional adducts were formed. The same was true for the incubation with a short oligonucleotide sequence (^5′^dCATGGCT), as shown in Fig. 29, left branch. The formation of monofunctional adducts **41a** and **41b** was due to the release of the pyridyl ligand **39-octreotide** or **39-RGD** (depending on the compound used) upon irradiation, followed by formation of the aqua species [Ru(η^6^-*p*-cymene)(bpm)(H_2_O)]^2+^ (bpm = 2,2′-bipyrimidine) **40**. This intermediate then reacted with one guanine present in the sequence, yielding the isomers **41a** and **41b**. On the other hand, when the solution containing the Ru–peptide conjugates and the oligonucleotide was intensively irradiated (9 J cm^–2^, 9 h irradiation), the bifunctional adduct **41c** appeared, due to consequent arene release (see Fig. 29, right branch). Encouragingly, the authors demonstrated the specificity of the ruthenium complex for guanine over other potential biological ligands present in octreotide such as histidine or methionine, since no interactions between the ruthenium complex and these amino acids were observed. Nevertheless, these promising results and targeted strategy still need to be verified in cell-based assays.

#### DNA intercalation and photo-cleavage

**Fig. 30:**
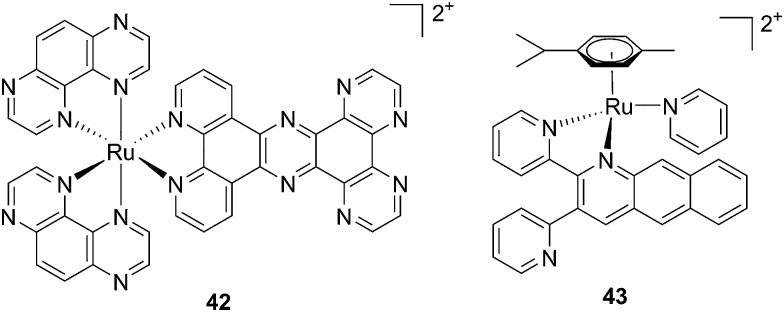
Structures of [Ru(TAP)_2_PHEHAT]^2+^ **42** studied for DNA intercalation and nicking by de Feyter and [Ru(η^6^-*p*-cymene)(dpb)(py)]^2+^ **43** synthesized by Wang, characterized by high wavelength absorption and ligand release.^95,96^

Covalent binding to guanine is not the only manner by which a (metal-based) drug can interfere with DNA replication. Different compounds have indeed been shown to interact with DNA in a non-covalent fashion. Flat aromatic structures (*e.g.* dppz or PHEHAT = 1,10-phenanthrolino[5,6-*b*]1,4,5,8,9,12-hexaazatriphenylene; TAP = 1,4,5,8-tetraazaphenanthrene; IPPBA = 3-(1*H*-imidazo[4,5-*f*][1,10]phenanthrolin-2yl)phenylboronic acid) are known to intercalate between two DNA bases. These DNA intercalative moieties place the metal in close proximity to the bases, facilitating direct photo-induced oxidation of guanines or DNA cleavage, for instance. To this end, de Feyter's group evaluated the effect of flat aromatic ligands on DNA conformation upon intercalation and upon photo-irradiation of [Ru(TAP)_2_PHEHAT]^2+^ (**42**) (Fig. 30).^95^ However, since no biological evaluation in cells or mice has been performed, only the important findings will be mentioned in this section. The authors could demonstrate that the main binding motif of the complex to DNA occurred *via* intercalation of the PHEHAT ligand. They could also highlight the importance of hydrogen bond-mediated TAP intercalation in DNA in the nicking activity upon visible light irradiation, since a decrease in nicking activity was observed when hydrogen bonds were prevented by urea. Nevertheless, urea treatment had no effect on the second type of DNA damage observed upon irradiation, namely adduct formation between the complex and DNA.

**Fig. 31:**
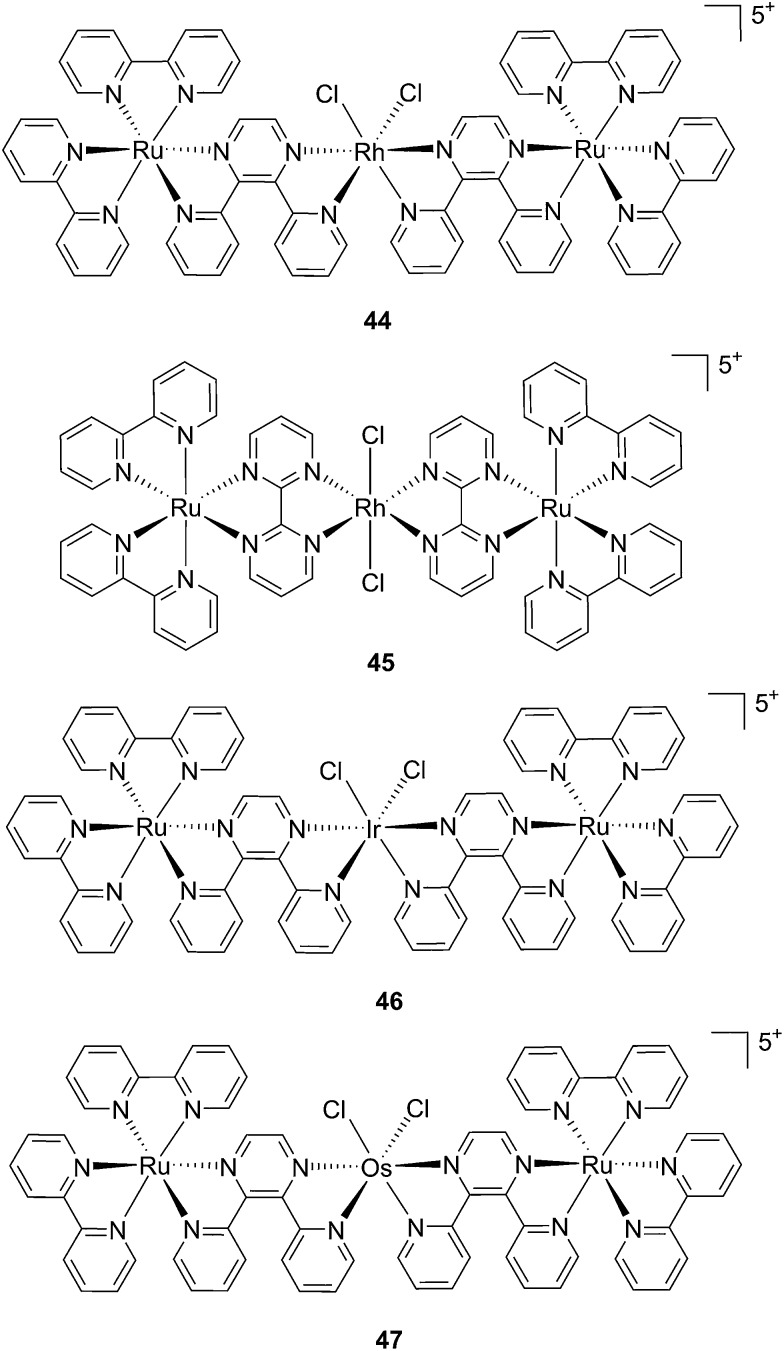
Structures of the mixed-metal supramolecular complexes; (**44**) [{(bipy)_2_Ru(dpp)}_2_RhCl_2_]^5+^, (**45**) [{(bipy)_2_Ru(bpm)}_2_RhCl_2_]^5+^, (**46**) [{(bipy)_2_Ru(dpp)}_2_IrCl_2_]^5+^, and (**47**) [{(bipy)_2_Ru(dpp)}_2_OsCl_2_]^5+^.^97,99^

Recently, Wang *et al.* succeeded to shift the absorption of a Ru complex to longer wavelengths in order to obtain ligand photorelease in the PDT window. At the same time, the authors achieved an increase of the lifetime of the excited state, which facilitates ^1^O_2_ production, thus combining PDT and PACT mechanisms. The authors undertook the latter by introduction of a 2,3-bis(2-pyridyl)-benzoquinoxaline (dpb) ligand into the structure of the Ru(ii) complex, whose delocalized π system was able to shift ^1^MLCT absorption to lower energy.^96^ The authors observed the formation of several photoproducts when [Ru(η^6^-*p*-cymene)(dpb)(py)]^2+^ (py = pyridine) (**43**) (Fig. 30) was irradiated with visible light (>400 nm) with the moieties (py) and (dpb) released in a 3.4 : 1 ratio. Moreover, the authors showed that the complex was a modest ^1^O_2_ generator with a ^1^O_2_ quantum yield of 0.25 in CH_3_CN. Further investigation of the direct effects on DNA revealed the photo-binding and photo-cleaving ability of the complex. These behaviors were subsequently examined in cell-based assays. After 4 h incubation in A549 cells, followed by 1 h irradiation at >400 nm and an additional incubation of 20 h in the dark, the complex showed enhanced toxicity after light irradiation, although with a moderate PI of about 7 (IC_50_ in the dark: 27.6 μM, upon light exposure: 4.0 μM). Brewer and co-workers also aimed to tune the absorption of the Ru(ii) complexes to lower energy wavelengths and to not be, at the same time, dependent on the presence of oxygen to achieve cell killing. To this end, they designed a mixed-metal supramolecular complex [{(bipy)_2_Ru(dpp)}_2_RhCl_2_]^5+^ (**44**, Fig. 31) containing two Ru(ii) centers to absorb visible light and one Rh(iii) atom.^97^ Complexes containing Rh(iii) have previously been shown to photo-cleave DNA.^98^ By agarose gel shift assays, the authors were able to characterize the structural requirements for the photo-cleavage process. First, they could show that the presence of rhodium in the molecule is needed for the DNA cleavage to occur. Indeed, when DNA was irradiated for 10 min at >475 nm, no cleavage was observed in the presence of the analog compounds lacking Rh(iii), both in the presence and absence of oxygen. Second, they could demonstrate that the process followed metal-to-metal charge transfer (MMCT) and not the ordinary MLCT process. To show this, the authors compared the DNA photo-cleavage efficiency of two analogs with inaccessible Rh(dσ*) and Ir(dσ*) orbitals, namely [{(bipy)_2_Ru(bpm)}_2_RhCl_2_]^5+^ and [{(bipy)_2_Ru(dpp)}_2_IrCl_2_]^5+^ (**45** and **46** in Fig. 31). As expected, both analogs failed to cleave DNA. The same group then tested the photo-triggered impact of their Ru–Rh mixed-metal complex on African green monkey kidney epithelial cells (vero cells) replication.^99^ The authors demonstrated that when cells were pretreated with **44** and exposed to >460 nm light, limited growth was observed up to 12 μM (conditions: 48 h incubation with compounds, then removal of the medium and 4 min irradiation at >460 nm, followed by 48 h recovery). At higher concentrations, cell death was observed. Interestingly, cell death was not observed with cells pretreated with the osmium analog complex **47**, shown in Fig. 31. This last observation highlighted the fundamental role of the Ru atoms in **44** in triggering cell death.

### Gene silencing (ODN strategy)

**Fig. 32:**
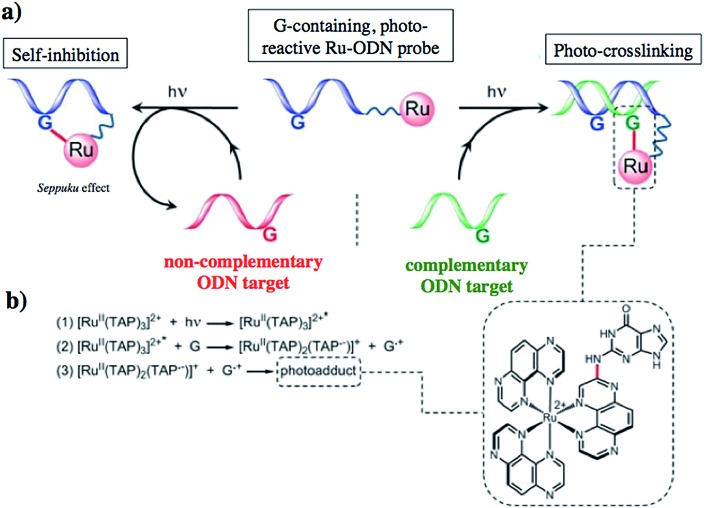
(a) Schematic representation of the Ru–ODN strategy, (b) explanation of the adduct formation. Adapted from ref. 102.

**Fig. 33:**
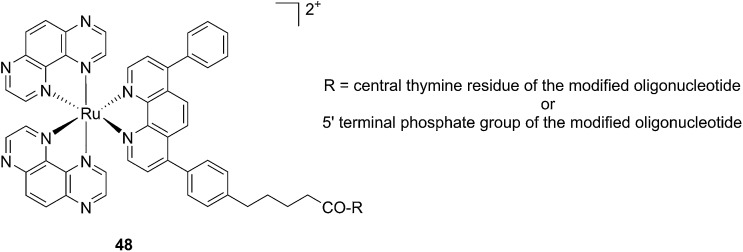
Structure of the [Ru(TAP)_2_dip]^2+^ conjugated to the ODN sequences.^101^

Another attractive approach to target DNA is the use of oligodeoxyribonucleotides (ODNs) to inhibit gene expression. ODNs can act on different kinds of targets, namely double-stranded DNA by triple helix formation (antigene strategy) or mRNA (antisense strategy). However, these strategies suffer from the low stability of ODNs, their ineffective delivery into cells and from the low affinity of the ODNs for the target sequence. To overcome these drawbacks, chemically modified ODNs have been investigated with different moieties, including ruthenium complexes. In the last few years, the group of Kirsch-De Mesmaeker focused its attention on the detection of nucleic acids using metal complexes.^100^ As an example, they employed highly photo-reactive Ru complexes to irreversibly crosslink ODNs to a DNA target sequence. A photoinduced electron transfer (PET) takes place between a Ru complex and the guanine present in close vicinity in the complementary DNA sequence. This results in covalent binding between the Ru complex and the guanine, forming a crosslink (see Fig. 32). The authors first examined the different geometric factors influencing adduct formation using [Ru(TAP)_2_dip]^2+^ (dip = 4,7-diphenylphenanthroline) (**48**, Fig. 33). They demonstrated that guanines at the 3′ side of the complementary strand, compared to the ruthenium complex anchoring position, are more favorable for the recombination of radicals formed by PET (irradiation settings: 1 h at 4 °C with a mercury/xenon lamp (200 W) using a filter (2M KNO_3_ solution)).^101^

**Fig. 34:**
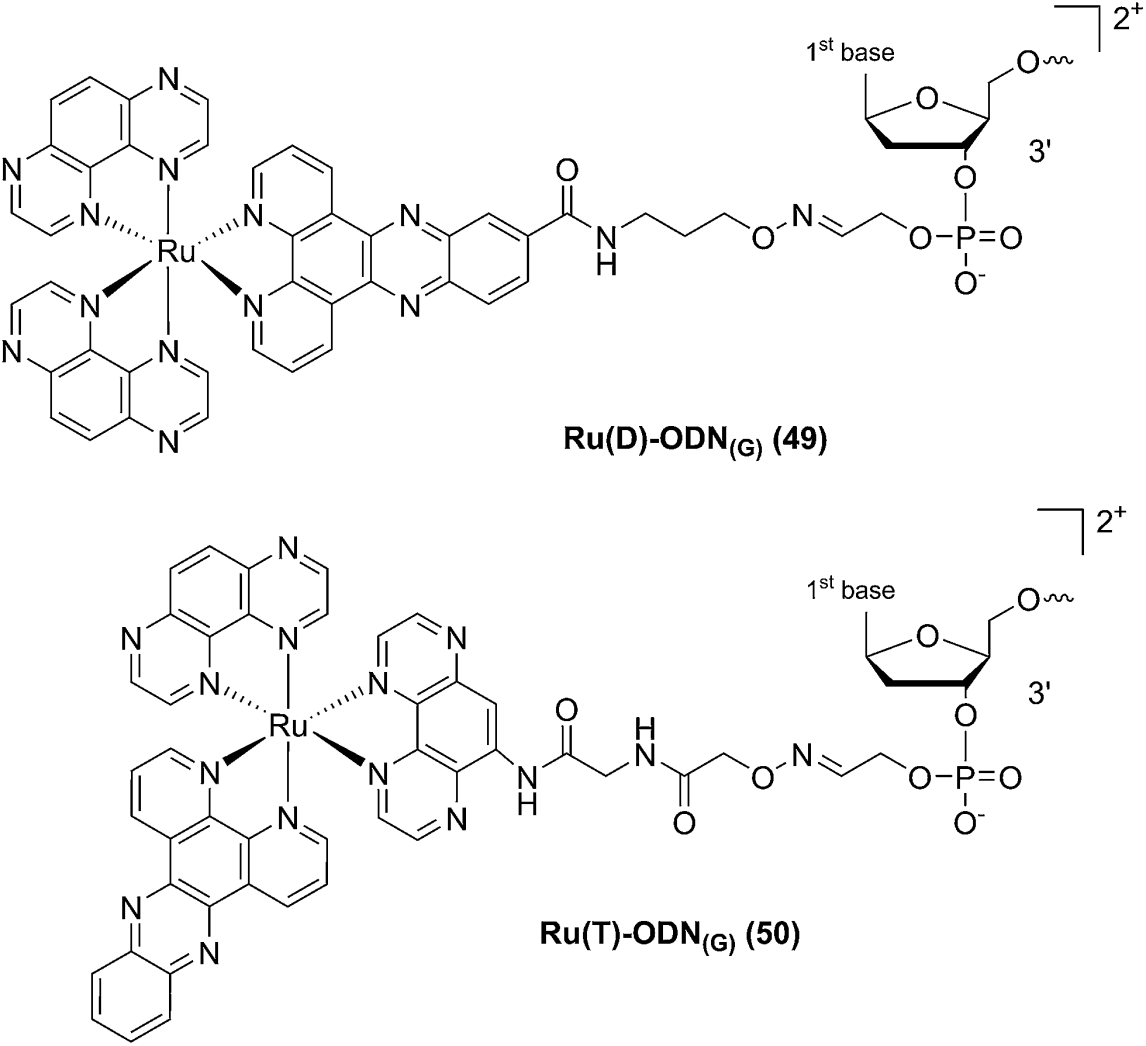
Structures of the Ru(ii) complexes with an ODN sequence coupled either on the dppz (**49**) or on the TAP (**50**) moiety.^102^

In a follow-up study, the same group explored the importance of the anchoring position of the Ru complex with respect to the ODN sequence. To do so, the photo-reactive polyazaaromatic complex [Ru(TAP)_2_(dppz)]^2+^ was coupled to the ODN sequence either *via* the dppz (*alias* Ru(D) (**49**) in Fig. 34) or the TAP moiety (*alias* Ru(T) (**50**) in Fig. 34). Adduct formation efficiencies and DNA interactions were evaluated. The authors found that both versions of anchored Ru complexes had the same DNA photo-ligation efficiency upon light irradiation (442 nm, 50 mW, 60 min) but, interestingly, they interacted with DNA in a different fashion. Whilst Ru(T) interacts by intercalation with the dppz ligand, Ru(D) interacts *via* TAP without intercalation. On one hand, dppz intercalation places the ruthenium center in direct contact with the guanine, favoring the PET process and back electron transfer. However, this geometrical conformation secludes the reduced TAP˙^–^ and oxidized G^+^˙, thus reducing the photo-crosslinking efficiency. On the other hand, the TAP interaction puts TAP˙^–^ and oxidized guanine in an optimal orientation for the photo-crosslinking reaction.^102^

**Fig. 35:**
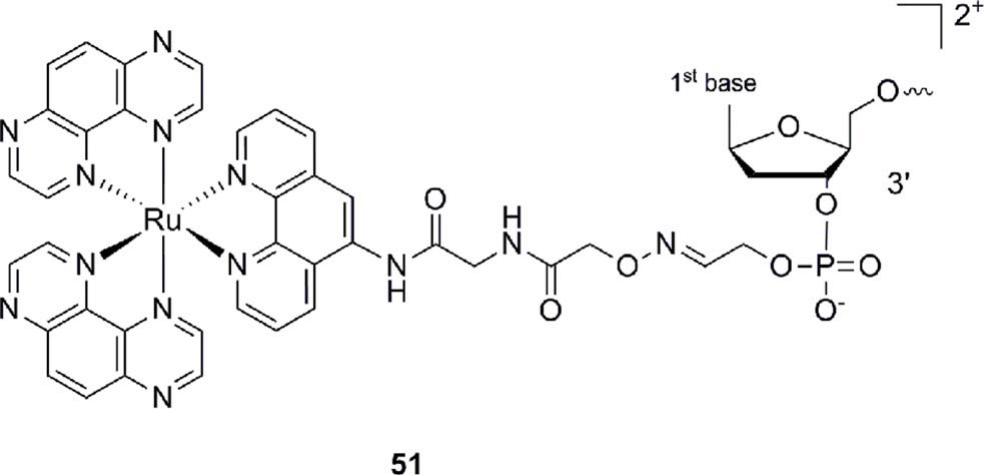
Structure of [Ru(TAP)_2_(phen)]^2+^.^103^

This Ru–ODN strategy was examined in cell-based assays.^103^ In their study, Delvenne and co-authors coupled the complementary sequence of the oncogene E6, stimulated after HPV16 infection and responsible for the silencing of p53, to a polyazaaromatic complex [Ru(TAP)_2_(phen)]^2+^ (**51**, Fig. 35). With this tool in hand, they tested the efficiency of the conjugate for impairing HPV16 positive cervical cancer cell (SiHa) proliferation upon visible illumination. They could demonstrate an irreversible crosslink between the target and the Ru-conjugated probe. The authors could not only show reduced cell proliferation (45–50% growth inhibition on SiHa cells, 24 h post illumination at 380–480 nm for 2 h 30), but also a restored amount of p53, which is the principal target of E6, as well as a reduced E6 protein level. The use of photo-reactive Ru complexes for conjugation to ODN sequences offers, in light of the studies presented above, an attractive tool in the field of gene silencing. On one hand, the presence of a metal complex can enhance cell delivery of the Ru–ODN due to the positive charge brought by the Ru complex. On the other hand, the affinity of the probe for the target is improved since an irreversible crosslink is induced upon light irradiation. Moreover, undesired non-specific interactions are avoided if the DNA target sequence is not present, since the complex is capable of auto-inhibition (the ‘seppuku effect’), thus eliminating any collateral inhibition or crosslink (see Fig. 32).

### Photo-release strategy

Modification of the activity of a compound can be achieved by masking the functional groups involved in the toxicity with a cleavable moiety, acting as a cage. The idea of using light-responsive cages to deliver biologically active compounds into living cells or organisms is extremely appealing, offering control over the cytotoxicity and improved cellular uptake thanks to the modulation of the lipophilicity or the insertion of a charge. However, to date, there are only a few examples reported in the literature. Those are described below.

#### Ruthenium complexes as caging moieties

**Fig. 36:**
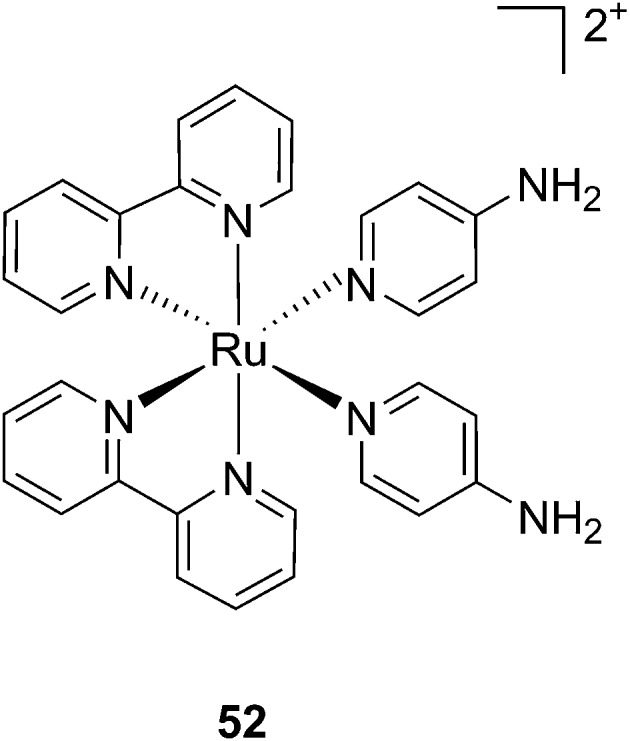
Structure of the Ru(ii) caged neuroactive 4-AP.^104^

The first study mentioned in this part of the perspective does not actually deal with cancer therapy, but presents for the first time the concept of using a Ru complex as a suitable cage for molecules bearing nitrogen atoms. Indeed, the [Ru(ii)(bipy)_2_] fragment was the first ruthenium cage used to release a molecule. Etchenique *et al.* caged the neurocompound 4-aminopyridine (4-AP) (**52**, Fig. 36), which is known to promote neuronal activity by blocking specific K^+^ channels.^104^ The authors were able to monitor the electrical activity of a neuron within an isolated ganglion after photo-release of 4-AP. First, as expected, they confirmed that the caged 4-AP did not change the electric pulse when kept in the dark. A similar behavior was found for the cage itself. Nevertheless, upon pulsed irradiation (pulsed Xe lamp, 0.5 J per pulse, low pass filter at 480 nm), a signal similar to the one of the free 4-AP was detected, demonstrating the photo-release of the neuro-compound. Of note, Etchenique and coworkers presented the release of other bioactive compounds upon light activation such as GABA,^105–107^ glutamate,^108,109^ nicotine^110^ and dopamine.^111^ However, this impressive work is not discussed herein since it is not related to anticancer research.

**Fig. 37:**
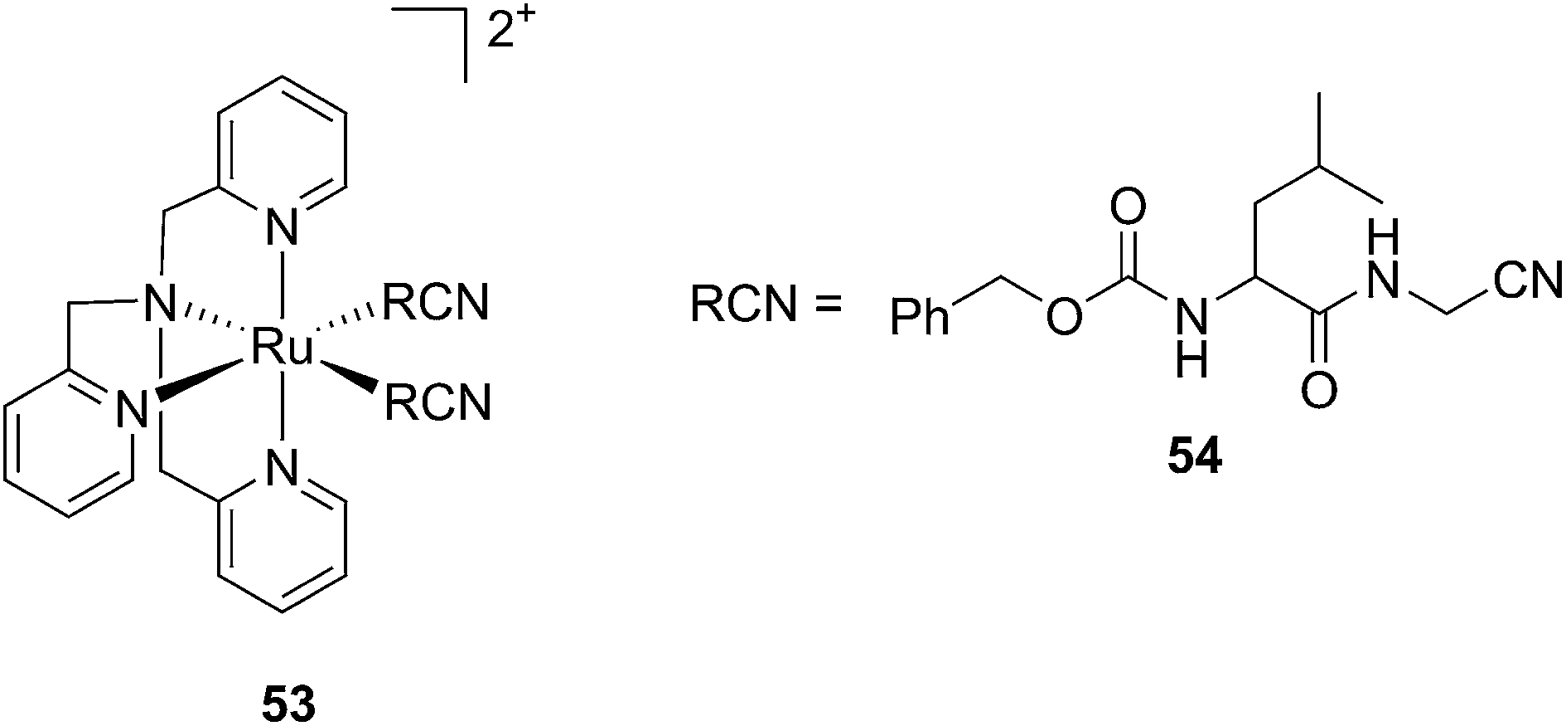
Structures of caged cathepsin K inhibitor (Cbz-Leu-NHCH_2_CN) (RCN).^112^

Kodanko and collaborators, in turn, considered the latter study and the opportunity offered by Ru complexes to act as an effective photo-caging group for nitriles. They synthesized a Ru(ii)tris(2-pyridylmethyl)amine complex **53**, functionalized with two molecules of a known cathepsin K inhibitor **54** containing a nitrile group (Cbz-Leu-NHCH_2_CN) (Fig. 37).^112^ In healthy tissue, cathepsin K is a proteinase secreted by osteoclasts to degrade bones. It was shown to be expressed by breast cancer metastasized to bones as well. The authors were able to demonstrate the inhibition of the enzymatic activity of cathepsin K upon light activation (365 nm for 15 min) even if only one molecule of the inhibitor was released (IC_50_ values of 5.6 μM in the dark *vs.* 63 nM upon light irradiation).

**Fig. 38:**
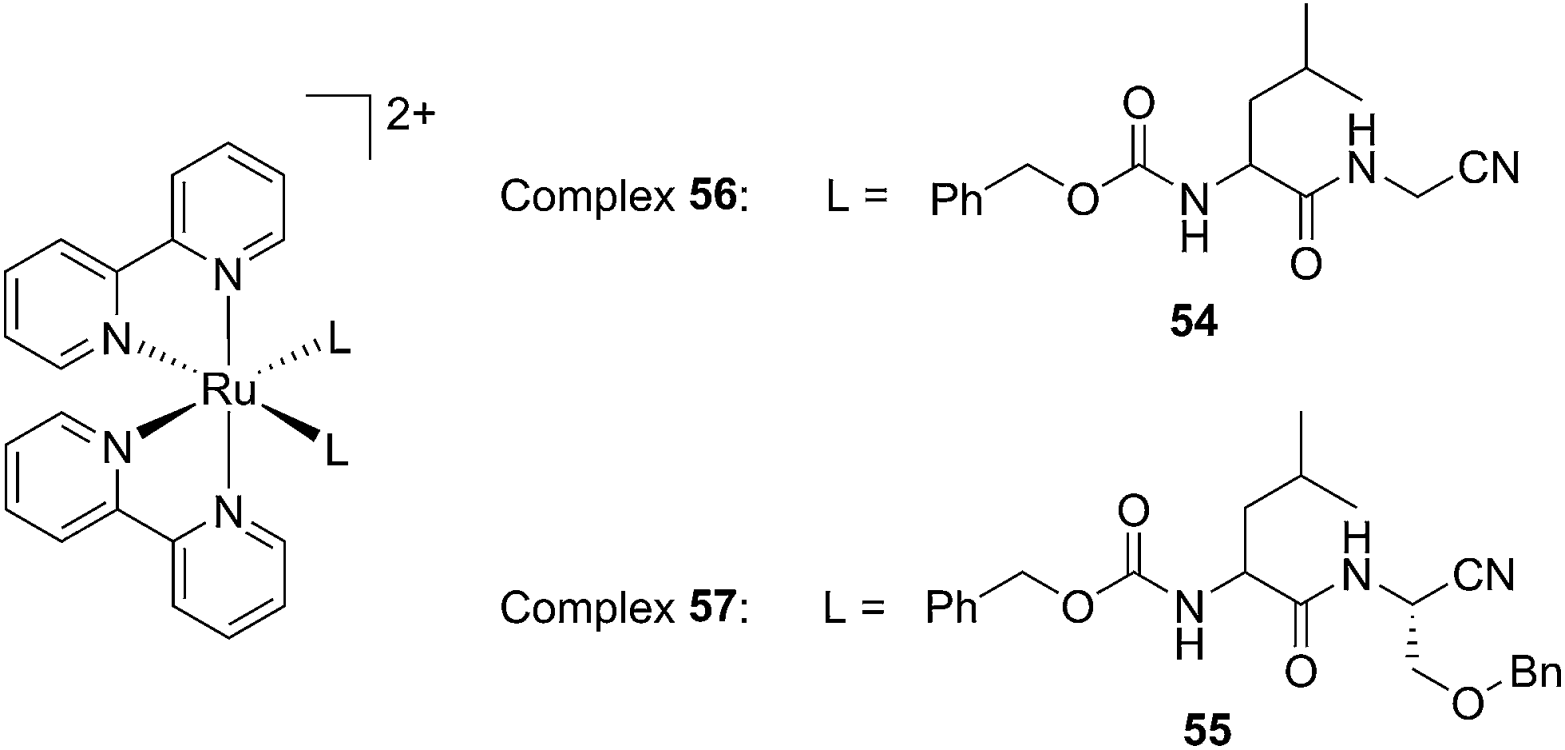
Structures of the Ru(ii) complexes containing cathepsin K inhibitors.^113^

Driven by these promising *in vitro* results, the same authors chose a different Ru cage and a second cathepsin K inhibitor **55**, yielding the compounds **56** and **57** reported in Fig. 38.^113^

**Fig. 39:**
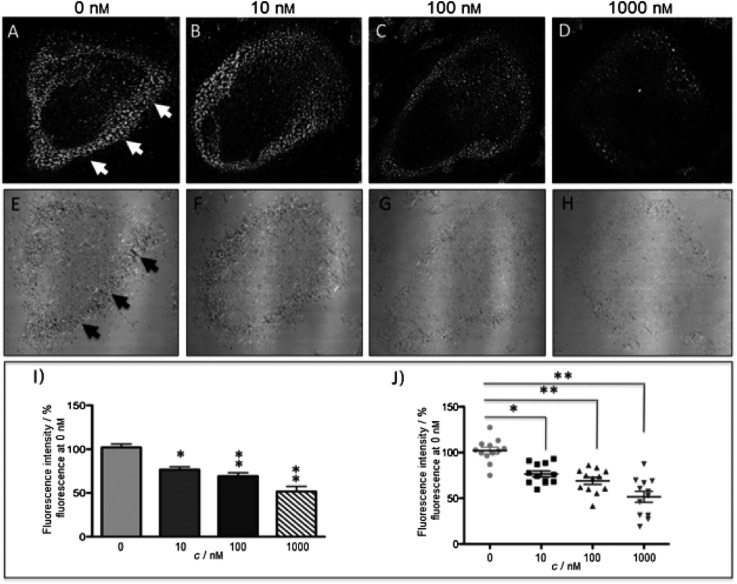
Confocal microscopy images of mouse osteoclast cells treated with **54**. Cells were pre-incubated with **54** (10–1000 nm) for 30 min at 37 °C in the presence of cathepsin B inhibitor CA074Me (1 mm). Cells were treated with the cathepsin K substrate Z-LR-4MbNA (0.25 mm) and nitrosalicylaldehyde (1.0 mm, a precipitating agent), leading to the release of 4MbNA (green fluorescent precipitate indicative of cathepsin activity, arrows). Cells were fixed and imaged with a confocal laser scanning microscope (Zeiss LSM 780) using a 40× oil immersion lens. For each of the conditions at least six images of individual osteoclast cells were acquired, and fluorescence intensity per osteoclast area was measured and quantified using ImageJ software (NIH). The intensity of green fluorescence is a direct measure of the quantity of hydrolyzed and precipitated substrate (A–D), also visible on DIC images (E–H). The quantified data are shown as column (I) and dot (J) plots; *p < 0.05, **p < 0.001. Results are representative of at least three experiments. Reproduced with permission from ref. 113. © 2014 Wiley-VCH Verlag GmbH & Co. KGaA, Weinheim.

**Fig. 40:**
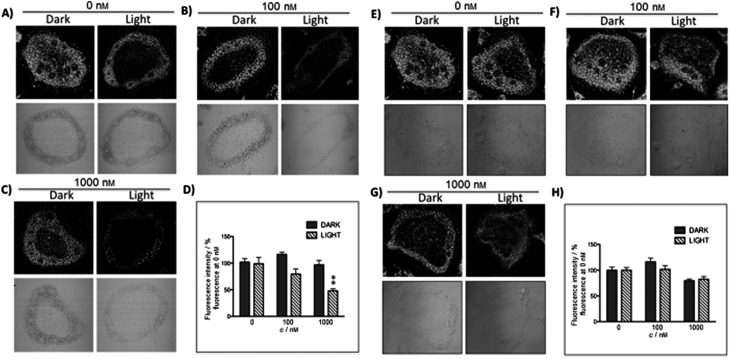
Confocal microscopy images of mouse osteoclast cells treated with the ruthenium-caged inhibitor **56** (A–D) or *cis*-[Ru(bpy)_2_(MeCN)_2_](PF_6_)_2_ (E–H). Cells were pre-incubated with either complex (0–1000 nm) for 30 min at 37 °C in the presence of cathepsin B inhibitor CA074Me (1 mm), then exposed to dark (no irradiation) or light (irradiation at 250 W, 395–750 nm) conditions for 15 min. Cells were treated with the cathepsin K substrate Z-LR-4MbNA (0.25 mm) and nitrosalicylaldehyde (1.0 mm, a precipitating agent), leading to the release of 4MbNA (green fluorescent precipitate indicative of cathepsin activity). Cells were fixed and imaged with a confocal laser scanning microscope (Zeiss LSM 780) using a 40× oil immersion lens. For each of the conditions at least six images of individual osteoclast cells were acquired, and fluorescence intensity per osteoclast area was measured and quantified using ImageJ (NIH) software as described for Fig. 39 above; **p < 0.001. Results are representative of at least three experiments. Reproduced with permission from ref. 113. © 2014 Wiley-VCH Verlag GmbH & Co. KGaA, Weinheim.

The new inhibitor **55** was chosen because of its better inhibition potency than **54** (reported IC_50_: 35 and 9 nM for **54** and **55**, respectively). Moreover, using the Ru(bipy)_2_ fragment as a cage, they could demonstrate that both nitrile ligands could be photoreleased. However, complex **57** required a longer exposure time to release the inhibitor (up to 60 min of irradiation) than complex **56** (15 min). Using the same experimental conditions (tungsten lamp, 250 W, >395 nm, H_2_O filter), **56** and **57** showed significantly enhanced inhibition activities compared to the parent inhibitors **54** and **55** (IC_50_ values are 36 nM and 28 nM, respectively). Complex **56** showed a dark to light IC_50_ improvement from 560 nM to 16 nM (PI = 35). The photo-activated inhibition of cathepsin K is twice as effective as the parent inhibitor alone, correlating with the two molecules of inhibitor released. In the case of complex **57**, the dark to light IC_50_ enhancement was from 2.2 μM to 25 nM (PI = 88). The light-triggered inhibition is in good agreement with the slow release rate of the second inhibitor molecule, reaching a similar value to the free parent molecule **55**. In order to verify that none of the drugs or photoproducts were toxic in cells, the authors tested the viability of Bone Marrow Macrophages (BMM) and PC3 cells after 30 min incubation with the complexes, followed by a dark environment or 15 min irradiation for **56** or 40 min for **57** and 24 h additional incubation. They were able to confirm that no toxicity was found in murine BMM or PC3 cells up to 10 μM for complex **56** and up to 1 μM for **57**. Since **56** showed the most promising features, the authors evaluated its enzymatic inhibition ability in a cell-based assay. Enzymatic activity in osteoclasts decreased by 25% and 50% when treated with 100 nM and 1 μM, respectively, either with **54** or with the photo-activated **56** (see the enzymatic inhibition studies in mouse osteoclasts in Fig. 39 for **54** and Fig. 40 for **56**). These findings suggest that the photo-released enzyme inhibitors can play a potent role in the treatment of diseases where increased enzymatic activity is observed, sparing normal activity in surrounding tissues. These studies widen the perspective on the use of Ru(ii) complexes as caging groups for the release of a large variety of biomolecules, from the pioneering Ru(ii)(bipy)_2_ fragment as a neurotransmitter releaser, to nitrile-based protease inhibitors.

**Fig. 41:**
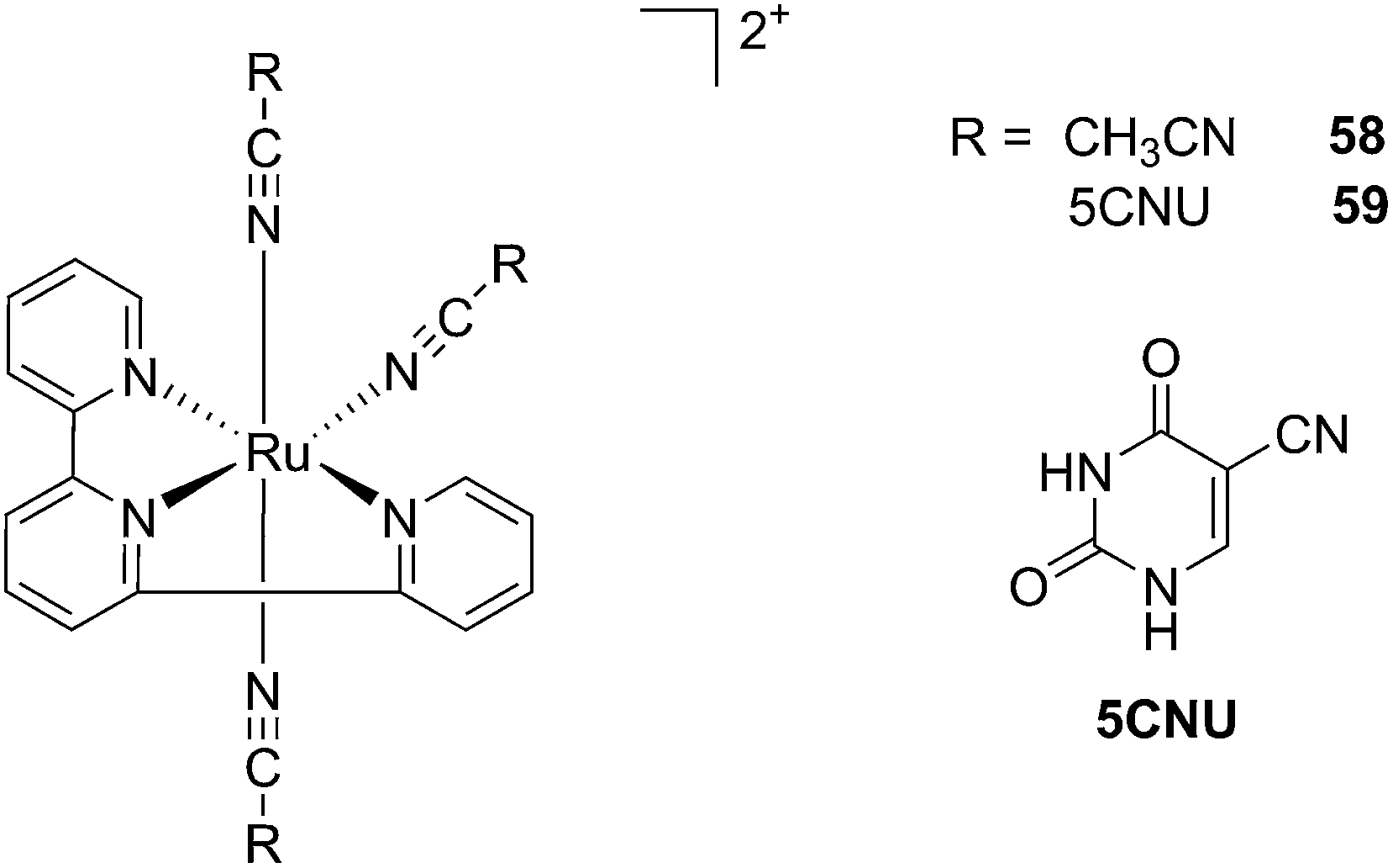
Structures of the Ru-inhibitor complexes synthesized by Turro.^114^

Kodanko and colleagues applied the Ru(bipy)_2_ fragment as a cage. However, compared to this, the Ru(ii)tpy fragment offers a lower energy ^1^MLCT absorption, fitting within the PDT window (600–850 nm). Turro *et al.* took this opportunity and designed a Ru(ii)tpy complex able to induce the release of 5-cyanouracil (5CNU), a known pyrimidine catabolism inhibitor, upon irradiation with visible light (>400 nm). Since it was shown that the bis-aqua Ru derivative can bind DNA, the following [Ru(tpy)(5CNU)_3_]^2+^ complex (**59**, Fig. 41) can potentially be used as a dual-action therapeutic agent.^114^

Indeed, the authors demonstrated that similarly to its analog complex bearing (CH_3_CN)_3_ (**58**, Fig. 41), the complex efficiently released the two axial ligands when irradiated with visible light (150 W Xe lamp housed in a Milliarc compact arc lamp housing), concomitantly producing the bis-aqua species. Only the latter photoproduct was then able to bind to DNA as observed by a reduction in the plasmid pUC19 mobility, when complexes **58** and **59** were irradiated for 5 or 15 min, respectively. However, extending these observations to cellular studies was revealed to be more challenging. Indeed, when human cervical cancer HeLa cells were treated with 100 μM of the Ru complexes for 2 h in the dark, followed by 1 h light irradiation, only complex **59** was shown to be capable of generating damage (no damage was observed in non-irradiated cells for **58** and **59**). Moreover, cells treated with 100 μM of free 5CNU showed the same extent of damage as for complex **59** upon light irradiation, coinciding with only one molecule of 5CNU being released. The latter observation was confirmed when the LC_50_ value of irradiated **59** matched the one of free 5CNU (156 and 151 μM, respectively). In both cases, the mono-aqua photoproduct formed was not able to bind DNA. Accordingly, no decreased mobility was observed in agarose gel shift assay. This was also confirmed by the absence of cytotoxicity upon irradiation in the case of **58** or increased toxicity for **59**.

#### Ruthenium complexes as photo-released drug candidates

**Fig. 42:**
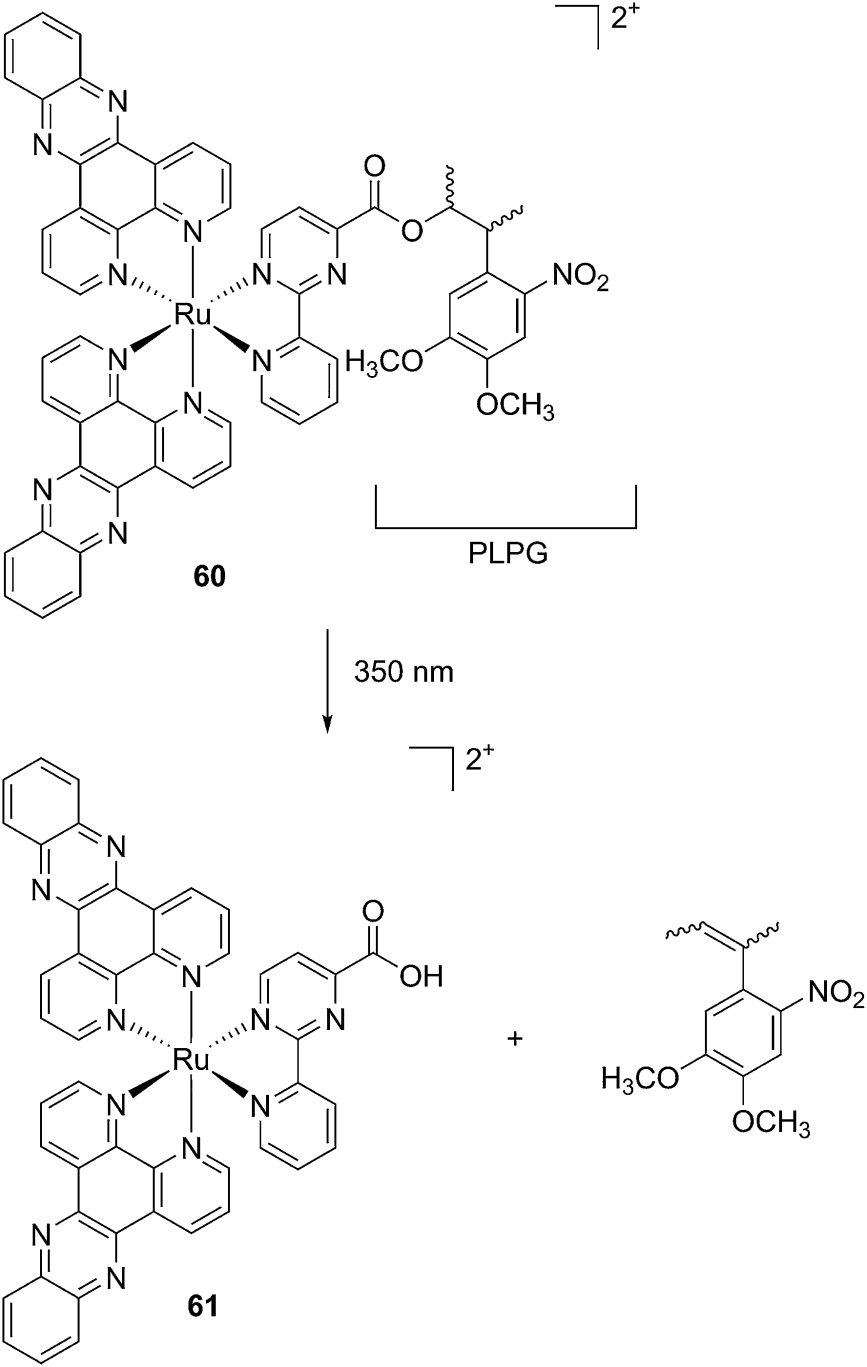
Structures of the caged Ru(ii) complex **60** and of the toxic photoproduct **61** which is released.^51^

The photo-cleavage of a cage to release bioactive components is not a novel strategy since this method has been successfully used to release small organic molecules.^115^ However, to the best of our knowledge, the specific release of a cytotoxic metal complex has never been reported before the work of our group. Indeed, we could recently successfully inactivate a previously characterized cytotoxic ruthenium complex (**61**)^116^ by attachment to a photo-labile protecting group (PLPG) (**60**, Fig. 42). UPLC-MS experiments confirmed that, upon UV-A exposure, the original complex was released from the PLPG. As previously suggested by SAR studies,^50^ we could demonstrate that caging reduced the toxicity of the Ru complex (IC_50_ > 100 μM in the dark) and that the original toxicity could be regained upon irradiation at 350 nm (2.58 J cm^–2^) (17 μM). This value coincides with the value of the original non-caged complex (**61**).^51^ We also investigated the fate of the Ru complexes by confocal microscopy. We could show that the caged complex was probably relocalizing from the cytoplasm and nucleoli (before light irradiation) to mitochondria, which were previously shown to be the preferential target of this complex.^116^ Our group recently demonstrated that such a concept could be applied to a rhenium(i) organometallic complex.^51^ Although there are a few advantages to use UV-A light as a trigger in the context of light-activated drugs, this type of light is only able to penetrate up to the derma, which protects tissues in the lower layers. Therefore, it would be of high interest to push the light activation of these drug candidates to the PDT or near-IR window.

## Conclusions

As shown in this perspective article, Ru(ii) complexes offer several opportunities as light-activated drug candidates. Although very promising *in vitro* results have been achieved so far, the lack of *in vivo* studies (to the best of our knowledge, there have only been three reported so far) undoubtedly does not allow for a full assessment of the suitability of such compounds in a clinical context. However, we are confident that more such studies will be reported in the near future, shedding light on the full potential of these compounds.

## Abbreviations

[9]aneS_3_: 1,4,7-Trithiacyclononane
^3^IL: Triplet intraligand
aPDT: Antibacterial PDT
ALA: Aminolevulinic acid
bipy: 2,2′-Bipyridine
biq: 2,2′-Biquinoline
bpm: 2,2′-Bipyrimidine
CALI: Chromophore-assisted light inactivation
DAPI: 4′,6-Diamidino-2-phenylindole
DLI: Drug to light interval
dmb: 4,4′-Di-methyl-2,2′-bipyridine
dmdop: 2,3-Dihydro-1,4-dioxino[2,3-*f*]-2,9-dimethyl-1,10-phenanthroline
dmphen: 2,9-Dimethyl-1,10-phenanthroline
dop: 2,3-Dihydro-1,4-dioxino[2,3-*f*]-1,10-phenanthroline
dpb: 2,3-Bis(2-pyridyl)-benzoquinoxaline
dpp: 2,3-Bis(2-pyridyl)pyrazine
dppn: Benzo[*i*]dipyrido[3,2-*a*:2′,3′-*c*]phenazine
dppz: Dipyrido[3,2-*a*:2′,3′-*c*]phenazine
DSSCs: Dye-sensitized solar cells
dtbb: 4,4′-Di-*t*-butyl-2,2′-bipyridine
en: Ethylenediamine
GSH: Glutathione
HR-CS AAS: High-resolution continuum source atomic absorption spectrometry
ICP-MS: Inductively coupled plasma mass spectrometry
IPPBA: 3-(1*H*-Imidazo[4,5-*f*][1,10]phenanthrolin-2yl)phenylboronic acid
ISC: Intersystem crossing
MLCT: Metal to ligand charge transfer
MMCT: Metal to metal charge transfer
ODNs: Oligodeoxyribonucleotides
PACT: Photoactivated chemotherapy
PCNA: Proliferating cell nuclear antigen
PDT: Photodynamic therapy
PET: Photoinduced electron transfer
Ph_2_phen: 4,7-Diphenyl-1,10-phenanthroline
PHEHAT: 1,10-Phenanthrolino[5,6-*b*]1,4,5,8,9,12-hexaazatriphenylene
phen: 1,10-Phenanthroline
phpy: 2-Phenylpyrydine
PI: Phototoxic index
PMP: 5-(3-Pyridyl)-10,15,20-triphenylporphyrin
PS: Photosensitizer
PTP: 5,10,15,20-Tetra(3-pyridyl)porphyrin
py: Pyridine
pydppn: 3-(Pyrid-2′-yl)-4,5,9,16-tetraaza-dibenzo[*a*,*c*]naphthacene
ROS: Reactive oxygen species
SAR: Structure activity relationship
SSBs: Single strand breaks
TAP: 1,4,5,8-Tetraazaphenanthrene
TPA: Two photon absorption
tpph: Tetrapyrido[3,2-*a*:2′,3′-*c*:3′′,2′′*-h*:2′′′,3′′′]phenazine
tpy: [2,2′;6′,2′′]-Terpyridine
